# Tissue and extracellular matrix remodeling of the subchondral bone during osteoarthritis of knee joints as revealed by spatial mass spectrometry imaging

**DOI:** 10.1038/s41413-025-00495-0

**Published:** 2026-01-26

**Authors:** Charles A. Schurman, Joanna Bons, Jonathon J. Woo, Cristal Yee, Qi Liu, Nannan Tao, Tamara Alliston, Peggi Angel, Birgit Schilling

**Affiliations:** 1 Buck Institute for Research on Aging, Novato, CA USA; 2 Department of Orthopaedic Surgery, University of California San Francisco, San Francisco, CA USA; 3 Bruker Daltonics Inc, San Jose, CA USA; 4 National Institutes of Health, Bethesda, MD USA; 5 Department of Pharmacology & Immunology, Medical University of South Carolina, Charleston, SC USA

**Keywords:** Bone, Diseases

## Abstract

Osteoarthritis (OA) is a degenerative skeletal condition marked by the loss of articular cartilage and changes to subchondral bone homeostasis. Treatments for OA beyond full joint replacement are lacking primarily due to gaps in molecular knowledge of the biological drivers of disease. Mass Spectrometry Imaging (MSI) enables molecular spatial mapping of the proteomic landscape of tissues. Histologic sections of human tibial plateaus from knees of human OA patients and cadaveric controls were treated with collagenase III to target extracellular matrix (ECM) proteins prior to MS Imaging of bone and cartilage proteins. Spatial MS imaging of the knee identified distinct areas of joint damage to the subchondral bone underneath areas of lost cartilage. This damaged bone signature extended underneath remaining cartilage in OA joints, indicating subchondral bone remodeling could occur before full thickness cartilage loss in OA. Specific ECM peptide markers from OA-affected medial tibial plateaus were compared to their healthier lateral halves from the same patient, as well as to healthy, age-matched cadaveric knees. Overall, 31 peptide candidates from ECM proteins, including Collagen alpha-1(I), Collagen alpha-1(III), and surprisingly, Collagen alpha-1(VI) and Collagen alpha-3(VI), exhibited significantly elevated abundance in diseased tissues. Additionally, highly specific hydroxyproline-containing collagen peptides, mainly from collagen type I, dominated OA subchondral bone directly under regions of lost cartilage but not areas where cartilage remained intact. A separate analysis of synovial fluid from a second cohort of OA patients found similar regulation of collagens and ECM proteins via LC-MS/MS demonstrating that markers of subchondral bone remodeling discovered by MALDI-MS may be detectable as biomarkers in biofluid samples. The identification of specific protein markers for subchondral bone remodeling in OA advances our molecular understanding of disease progression in OA and provides potential new biomarkers for OA detection and disease grading.

## Introduction

Osteoarthritis (OA) is the most common joint disease worldwide with the prevalence of OA increasing with age such that around half of all women (48.1%) and nearly one third of men (31.2%) aged 65 years and over^1,2^ suffer from this debilitating joint disease. The future burden of musculoskeletal diseases is only expected to escalate in the coming decades as lifespans increase and more individuals become affected by comorbidities and other risk factors for OA, such as obesity, hypertension, dyslipidemia, dementia, and Alzheimer’s disease.^3,4^ Given that osteoarthritis and its risk factors all increase with age, we sought to understand more deeply the spatial progression of molecular changes throughout joint tissues in patients with clinically diagnosed OA and in non-arthritic individuals.

The relationship between subchondral bone and articular cartilage changes in OA, with well-documented progressive changes in subchondral bone remodeling, mineralization, vascularity, and innervation that parallel cartilage degeneration.^5,6^ In the early stages of OA, subchondral bone undergoes increased bone resorption, but at later stages, deregulated bone remodeling leads to increased subchondral bone density.^5–7^ In addition to the thickening of the subchondral bone plate, OA is often accompanied by microarchitectural changes in trabecular bone and bone marrow lesions.^8,9^ Indeed, clinical magnetic resonance imaging (MRI) identifies early changes to subchondral bone as predictors of later stage joint pain and joint replacement, even before detectable changes in the overlying cartilage^10^ These observations motivate the search for diagnostics or interventions targeting subchondral bone to detect and retard OA progression, prior to the loss of articular cartilage, while the disease may still be reversible.^11^ However, many questions remain about the cellular and molecular targets for such interventions. Multiple cell types, including osteoblasts, osteoclasts, osteocytes, mesenchymal progenitors, and others, have been implicated in the subchondral bone changes in OA. In addition, aging, OA, and diseases such as type II diabetes cause changes in collagen post-translational modifications, including prolyl hydroxylation, glycation, and others^12,13^ tied to alterations in bone matrix material properties.^14,15^ In order to understand the loss of subchondral bone and cartilage homeostasis in OA, an unbiased spatial analysis of the proteomic changes in each component of the multi-tissue joint environment is needed. The complex nature of the ECM within bone and cartilage including their intrinsic properties have long obstructed proteomic profiling that has been successfully applied in most other tissues.

Matrix-Assisted Laser Desorption/Ionization - Mass Spectrometry Imaging (MALDI-MSI) is a rapidly advancing technology that allows for the direct detection of analytes in their spatial location directly from tissue sections. Initially introduced in the 1990s, MALDI-MSI has been applied in pathology research by providing an alternative to traditional histopathologic and immunocytochemical stains by directly imaging target proteins.^16,17^ Since its inception, MALDI-MSI has been used in several disease and tissue contexts including studies on neurodegenerative diseases and cancers, among others.^18–21^ This technology does not only comprise peptide-based spatial imaging but also MS Imaging of small molecule analytes, such as lipids, metabolites, and even large macromolecular glycan structures.^22–24^ The application of MALDI-MSI in the field of biomedical research, along with other mass spectrometry imaging technologies, such as water-assisted laser desorption ionization (SpiderMass)^25^ and parallel technologies like laser-captured microdissection,^26^ opened new frontiers by enabling the identification of possible new diagnostic compounds or biomarkers in place in their direct biological microenvironment.^27^

In previous years, investigators started to utilize MALDI-MSI to investigate the molecular landscape of OA, however, with a specific focus on lipids and glycans in cartilage and synovial tissues.^28–30^ Recently, MALDI-MSI was employed to investigate the lipid profiles and their distributions in the synovial membrane and infrapatellar fat pad of human OA patients, revealing the importance of lipid species in the development and regulation of inflammatory processes in OA, while others have extended these lipid and metabolite analyses to articular cartilage.^31–33^ Recently, spatial profiling of the subchondral bone has been achieved for lipids, expanding MALDI-MSI to mineralized tissues.^34–37^ In one recent application by Vandenbosch et al., MALDI-MSI was able to detect amounts of target lipids and small molecule targets in bone tissue at a doped amount of 2.5 pg.^38^ Other investigators utilized MALDI-MSI to study N-glycans in the subchondral bone of the knee in OA and identified specific glycans within subchondral trabecular bone that were upregulated in different joint quadrants that they project correspond to areas of altered mechanical loading with disease.^30,39,40^ While the latter studies represent advancements in the use of spatial glycan imaging in subchondral bone, to date very few studies attempted imaging of protein structures^30,41,42^ of joint tissues targeting the synovium and articular cartilage in human and equine samples.

The highly complex secondary and tertiary structure and the crosslinked nature of the bone ECM presents a technical hurdle in the application of MS Imaging for proteins and peptides to study subchondral bone changes in OA. However, recent significant advancements in the utilization of enzymatic protein digestions proved potent in the study of other ECM dense tissues and diseases such as breast, lung, and prostate cancers as well as in infarcted cardiac tissues.^20,21,43,44^ Collagenase III, also known as matrix metalloproteinase-13 (MMP-13), is a potent proteinase with a broad spectrum of activity targeting various ECM proteins, including collagen types I, II, and III. Homeostatically, this enzyme is involved in the degradation and turnover of the ECM, essential in processes such as skeletal development and remodeling, rendering it an ideal enzymatic choice to prepare bone and joint tissues for protein-based MALDI-MSI. Additionally, using collagenase instead of a more broadly active enzyme, such as trypsin, targets our analysis to relevant ECM proteins and eases computational demands. Future spatial proteomic works will utilize newer techniques, such as “imaging parallel reaction monitoring” (iprm) to deconvolute the dense and complex spectra produced by tryptic digestion for spatial proteomics.^45^

**Fig. 1:**
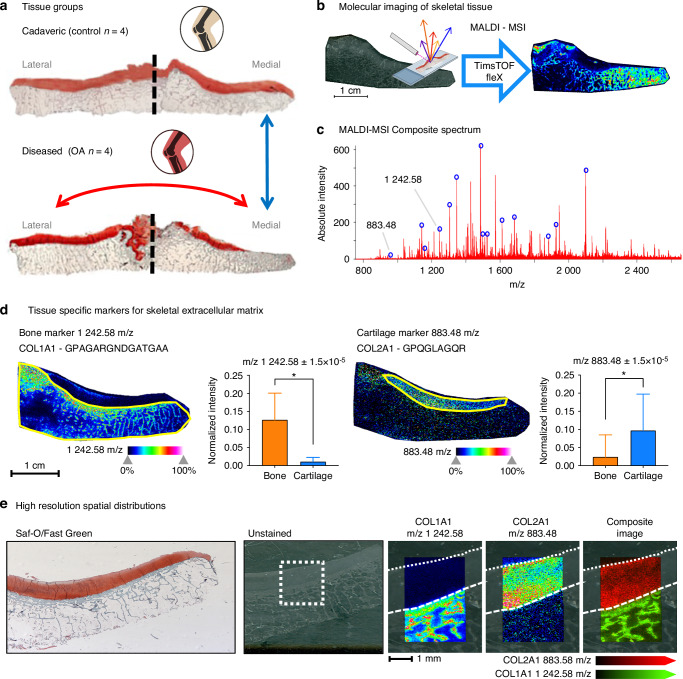
Arthritis of the Tibial Plateau and Spatial Proteomic Imaging. **a** non-arthritic cadaveric donors without joint disease and OA patients undergoing knee replacement that were age, sex, and BMI matched are stratified by their clinical OARSI (arthritis) grade. Safranin-O/Fast Green staining showed preserved proteoglycans in controls (top) but medial cartilage loss in OA (bottom). **b** Extracellular matrix proteins were profiled by MALDI-MSI on a timsTOF fleX (Bruker), enabling spatially resolved molecular imaging. **c** Composite spectra from medial and lateral tibial plateaus of control and OA samples revealed >3 000 peaks, supporting tissue-specific mapping of m/z features. **d** In control medial tibia, Collagen α1(I) localized to bone and Collagen α1(II) to cartilage, consistent with histology (yellow outlines). Quantitative analysis confirmed enrichment of each marker in its respective tissue (*P < 0.05). **e** High-resolution (20 µm) imaging at the bone–cartilage junction resolved distinct peptide signals: COL1A1 (m/z 1 242.58) in trabecular bone and COL2A1 (m/z 883.48) in cartilage, highlighting the precision of spatial proteomics

In this study, we spatially investigated the extracellular protein content and ECM remodeling of human end-stage OA of the knee, to demonstrate the first use of enzymatic digestion of formalin-fixed paraffin-embedded (FFPE) knee joint tissues (mounted on a microscopic ‘slide’) from non-arthritic cadaveric control donors (*n* = 4) and age-matched OA patients (*n* = 4) for MALDI-MS Imaging of ECM proteins (Fig. 1a). In addition, these samples were prepared for serial glycan and ECM protein imaging,^46,47^ demonstrating the general ability to complete multi-omics imaging on the same slides of human joint tissues composed of both bone and cartilage regions. Our experimental strategy utilizing collagenase enzymatic digestion (followed by spatial mass spectrometry imaging) allowed for an “untargeted” assessment of a broad range of ECM and ECM-associated proteins in bone and cartilage. This allowed for the visualization and classification of differences in ECM protein composition in OA and during healthy aging, including differences in hydroxy-proline post-translational modifications, in an untargeted manner without the use of antibodies. Through the identification of disease-regulated ECM proteins in OA, molecular mechanisms aggravating disease progression, potentially originating in the subchondral bone, may be spatially visualized for the first time to provide much-needed novel disease markers that may be utilized to track, or even predict, osteoarthritis in humans.

## Results

### MS imaging and spatial proteomics of skeletal tissues in the knee

This study analyzed spatial molecular proteomic changes in the medial tibial plateau of the knee of a small prospective cohort of patients with medial osteoarthritis (OA) (*n* = 4) compared to either the medial tibial plateau of non-arthritic cadaveric joints (control) (*n* = 4) or the lateral tibial plateau of the same OA patients that did not show extensive cartilage loss via Safranin/Fast green histological stain (Fig. 1a). This prospective OA cohort was composed of only male donors as were available through the Department of Veterans Affairs Medical Center (San Francisco, CA, USA). All donors received total knee arthroplasty at the Department of Veterans Affairs Medical Center. Non-arthritic control tissue was acquired through the Willed Body Program at the University of California San Francisco and selected to match OA donors in sex, BMI, and age, resulting in a cohort of human tibial plateaus that were stratified by their Osteoarthritis Research Society International (OARSI) score (Figs. S1 and 2, Table S1).

Given the complexity of osteoarthritis, and the need for improved molecular markers for the disease, we sought to utilize MALDI-MSI to investigate changes to the ECM in both subchondral bone and cartilage. Here, we demonstrate molecular imaging of proteomic data from knee joint tissues composed of both cartilage and bone regions using MALDI-MSI (Fig. 1b). Given that MALDI-MS Imaging studies of collagen type proteins in bone have not previously been reported, we first verified that the enzymatic approach using collagenase III could successfully provide relevant details about the skeletal ECM. From a representative non-arthritic, cadaveric control, 33 234 individual MS1 mass spectra, one for each imaged spot, across the entire tissue section at a spatial resolution of 120 μm (laser step size). Averaging the spectra for all imaged pixels demonstrated a composite spectrum representative of the entire tissue (Fig. 1c) with over 3 000 distinct individual peaks, each representing specific molecular features (peptides) with distinct mass-to-charge (m/z) ratios. Machine learning approaches on the top 500 most intense peaks were used to segment proteomes across the tissue (Fig. S3).

To determine how well the segmented proteins corresponded to actual tissue types, we investigated whether known protein markers of bone and cartilage would be preferentially located in these automatically identified regions (Fig. 1d). Proteolytic peptides from Collagen alpha-1(I) and Collagen alpha-1(II), identified in studies of other tissue types,^20,43,48^ were used as markers for bone and cartilage tissue within the non-arthritic sample, respectively. The bone marker protein Collagen alpha-1(I) and its corresponding peptide G^317^PAGARGNDGATGAA^331^ with m/z at 1 242.58, showed an intense signal predominantly in the ‘subchondral bone’ region (yellow outline, left), with almost no signal detected in the ‘cartilage’ region (yellow outline, right). Quantitatively, the pixel spots within the bone region had a significantly higher mean pixel intensity for the feature at m/z 1 242.58 than the collection of pixels within the cartilage area by*t*-test (*P* < 0.000 1). Conversely, the cartilage marker protein Collagen alpha-1(II) and its peptide G^982^PQGLAGQR^990^ with m/z at 883.48, was most intensely abundant in the identified cartilage region with little to no signal intensity within the bone region. Compared via *t*-test, the generated cartilage region also showed a significantly elevated mean pixel intensity (*P* < 0.000 1) at this m/z compared to the bone regions. The segmented tissue regions corresponding to cartilage and bone also match well to histopathology via Safranin-O/Fast Green stains on serial sections of the tissues (Fig. S2b). To more clearly ascertain that MALDI-MSI can discern between bone and cartilage on the molecular level, a smaller region of the bone-cartilage osteochondral junction was imaged at an improved spatial resolution using a laser step size of 20 μm (Fig. 1e), which (in our system) was the step size matching the minimal laser ablation area. At this improved higher spatial resolution, it was evident that the signal from the Collagen alpha-1(I) peptide G^317^PAGARGNDGATGAA^331^ at m/z 1 242.58 emerged uniquely from the trabecular bone. In contrast the peptide from Collagen alpha-1(II) G^982^PQGLAGQR^990^ with m/z 883.48 was detected specifically in the upper cartilage region. Figure 1e highlights the distinct spatial distribution of the bone and cartilage markers, and the two areas display distinct separation when visualized in a composite image. To solidify this finding, additional unique peptides from Collagen alpha-1(I) and Collagen alpha-1(II), G^626^PAGERGEQ^634^ with m/z at 900.40 and G^999^PSGEPGKQGAP^1010^ with m/z at 1 097.63 respectively, are also compared across the bone and cartilage regions and showed similarly significant differences in mean pixel intensity between bone and cartilage areas (Fig. S4A, B) providing distinct spatial features that correlate to the tissue types. These higher resolution settings (20 micron laser step size) revealed exciting spatial distributions for multiple features discernable with individual bone trabecula (Fig. S4C) – morphologic features that are themselves typically only at an average width of 200 microns. These spatial patterns reveal regions of high signal intensity specifically along the edges of trabecular bone for some m/z features, such as m/z 1 943.91, while others show the most intense signal in the innermost portions of trabecula, e.g. at m/z 1 242.58 and m/z 1 406.71. While these specific peptide markers illustrate almost a binary delineation between bone and cartilage tissue regions, other unexplored markers such as glycans or other ECM proteins can reveal even further granularity or gradients across the bone-cartilage osteochondral junction.

**Fig. 2:**
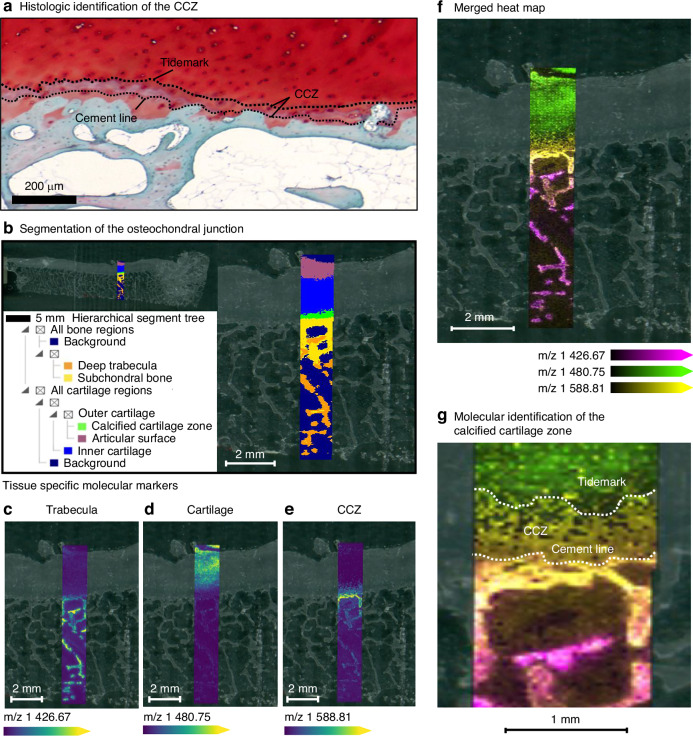
High-resolution MALDI-MSI of the osteochondral junction. **a** Safranin-O/Fast Green staining of the tibial plateau at higher magnification highlights cartilage tidemark, cement lines, and the calcified cartilage zone (CCZ). High-resolution MALDI-MSI (20 µm) was performed across cartilage, osteochondral junction (OCJ), and subchondral bone. **b** Automated segmentation identified distinct layers of cartilage, bone, and multiple OCJ subregions (green, yellow). **c**, **d** Discriminating feature analysis revealed tissue-specific markers that described these new tissue regions including for trabecular bone (m/z 1 426.67) and cartilage (m/z 1 480.75) **e** Specific features existed that revealed unique spatial distribution for the region in between bone and cartilage (m/z 1 588.81). **f**, **g** Overlay of feature heatmaps demonstrated matrix composition gradients and spatially distinct regions corresponding to histologic landmarks including cement lines, tidemark, and CCZ as is seen in histologic staining

Further imaging at higher resolution (20-micron laser step size), we sought to capture finer gradations of tissue compositional change across the osteochondral junction from the outer most cartilage surface deep into the trabecular bone. A well-known feature of the transition from cartilage to bone in the osteochondral junction is the calcified cartilage zone (CCZ)^49^ that lies in between the cartilage tidemark and the subchondral bone cement lines. These features were easily seen in histologic Safranin-O/Fast Green stains (Fig. 2a). A thin strip of tissue, 2 mm in width, was scanned with MALDI-MSI from the cartilage surface to the bottom depth of trabecular bone in the tibial plateau at 20 μm laser step size. Segmentation in SCiLS within this region (Fig. 2b) identified at least three distinct regions of cartilage including the outer articular surface layer (purple), internal cartilage (blue), and a lower outer boundary region separating cartilage from the subchondral bone (green) at the osteochondral junction, as indicated by the separation levels in the hierarchical clustering map. Subchondral bone separated into two distinct levels composed of an area of tissue containing the subchondral bone plate and adjacent trabecula (yellow) and a second region of deeper trabecular bone (orange). Discriminating feature analysis in SCiLS identified multiple m/z features that are characteristic of these different regions. For example, a marker specific to trabecular bone was found at m/z 1 426.67 (Fig. 2c) and a marker for cartilage was found at m/z 1 480.75 (Fig. 2d). Notably, features existed that had unique spatial distribution for the lower cartilage boundary region in between bone and cartilage at the osteochondral junction, such as the feature at m/z 1 588.81 (Fig. 2e). In order to fully appreciate the gradient of compositional change across the osteochondral junction, the spatial heatmaps for these selected m/z features are overlaid (Fig. 2f), which shows a more gradual transition in molecular species through the tissue depth than the segmented regions alone. Investigating the osteochondral junction at higher magnitude (Fig. 2g), the overlap of feature heatmaps revealed a region of tissue with strikingly similar delineation as the CCZ, where the most abundant intensity of the species at m/z 1 588.81 appeared in an area matching the ‘cement line’ boundaries found in histological analysis. In addition, the species at m/z 1 480.75 – a feature descriptive for cartilage – appeared in a region where the ‘cartilage tidemark’ was found histologically. In the future, further analysis and targeted LC-MS/MS will be needed to confirm the molecular identities of these additional specific molecular features. Currently, as we described above, the ability of our MALDI-MSI techniques to spatially visualize m/z features that uniquely match histologic features, even at small transitional areas like the calcified cartilage zone (CCZ), demonstrated the capability and premise of this technology for musculoskeletal biology.

### Subchondral bone remodeling in osteoarthritis

**Fig. 3:**
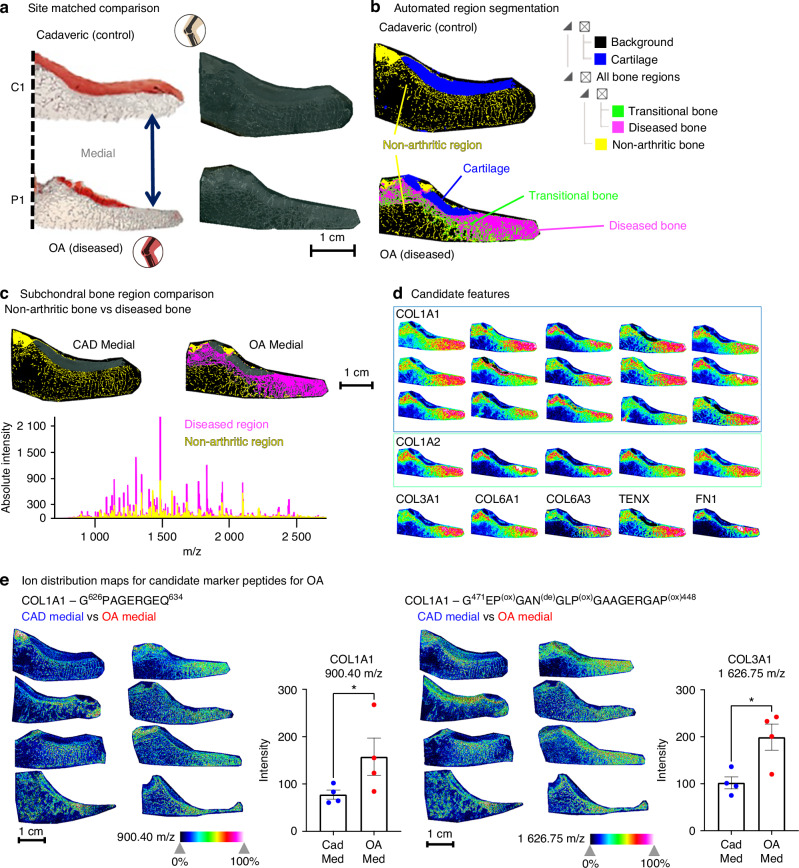
Site-matched comparison of OA vs. cadaveric medial tissues—candidate selection and validation. **a** Medial sections from cadaveric controls were compared with site-matched OA tissues. **b** Hierarchical segmentation of the top 500 m/z features identified cartilage (blue) and three bone subtypes: healthy bone (yellow), transitional layer (green), and diseased bone beneath cartilage loss (magenta). **c** Comparing healthy bone (yellow, present in both groups) with diseased bone (magenta, OA only) showed increased MALDI-MS peak intensities in diseased regions. Pairwise ROC analysis identified 31 discriminatory features (AUROC ≥ 0.85). **d** Candidate features from this analysis were validated in the full cohort (*n* = 4 per group), revealing significant OA-associated increases, including peptides from Collagen α1(I) (m/z 900.40) and Collagen α1(III) (m/z 1 626.75). *P < 0.05; see Table S2

Though some markers for cartilage degeneration can be detected in synovial fluid or in circulation, including small metabolites, lipids, inflammatory molecules, and by-products of proteoglycan and peptide catalysis,^50^ less is known about markers for bone degeneration. Since we determined that MALDI-MSI can accurately identify tissue type and tissue specific protein markers in aged, non-arthritic bone and cartilage at high spatial resolution, we applied this technique to investigate the molecular landscape defining OA to potentially uncover novel markers of disease. We used a candidate-validation approach to first identify markers in a representative pairwise comparison between tissues for both the medial, non-arthritic cadaveric control group and the medial, OA cohort and then “validated” these markers using the full donor cohort. First, one medial, non-arthritic cadaveric tibial plateau was used as a control and was compared to one site-matched OA, or diseased, medial tibial plateau (Fig. 3a). Acquired MALDI-MSI data from the representative control, C1, and the first OA patient, P1, were simultaneously loaded into the same data processing workspace. This resulted in a combined data set of 59 084 individual spectra. Automated region segmentation (Fig. 3b) for the top 500 most intense peaks from the combined dataset resulted in identification of multiple tissue regions. Similar to the segmentation processes for control tissue, the segmented regions captured the cartilage regions and identified regions of non-arthritic subchondral bone in both control cadaveric tissue and in the deeper trabecular bone of the OA patient. Interestingly, this “non-arthritic” region existed in both groups, the cadaveric control and OA individuals. The non-arthritic regions in the OA patient indicated a molecularly more “healthy”-appearing bone region, that was distinct from other bone regions revealed in the right half, or outer portion, of the medial tissue from OA patient P1. In that specific area, where cartilage deterioration and arthritis damage were most severe, we identified a thin transitional layer of bone in the OA patient (Fig. 3b, indicated in green) that separated the remaining healthier bone from a more profoundly impacted area of diseased arthritic tissue (Fig. 3b, indicated in magenta).

To investigate the molecular differences between all non-arthritic bone (Fig. 3b, yellow), including non-arthritic bone from the OA patient, and the OA diseased bone spatial regions (Fig. 3c), and to generate candidate features for comparisons between the two groups, we applied a Discriminating Feature Analysis that utilized a receiver operating curve (ROC) scoring algorithm.^51,52^ The ROC curve analysis indicated how well the spatial distributions of individual m/z features matched or predicted the various bone regions and their spatial boundaries. We identified 31 candidate m/z features (Table S2) that discriminated the non-arthritic and diseased spatial regions when requiring a statistical threshold with an ‘area under the receiver operating curve’ (AUROC) of >0.85. These m/z features represented the peptides that best differentiated between arthritic and non-diseased bone tissue across the representative tissue samples from cadaveric controls and OA patients. Candidate lists include peptides derived from collagen type I (Collagen alpha-1(I), Col1a1) and Collagen alpha-1(II), Col1A2) and collagen type III (Collagen alpha-1(III), Col3a1), see Table S2.

To assess whether candidate features could serve as potential osteoarthritis markers, the mean pixel intensity of each feature (m/z) was used as a quantitative metric and compared from defined regions of tissue (Fig. S5) containing diseased subchondral bone from each individual OA patient (*n* = 4) and non-diseased tissue from each cadaveric control (*n* = 4). Signal from each candidate feature was exported for pixels within these ROIs to avoid the transitional bone and non-arthritic bone subtypes in OA samples and used to evaluate each candidate feature’s mean pixel intensity for each individual patient or control donor. The mean pixel intensity for each candidate feature was compared between the site-matched cadaveric controls and the medial OA samples to determine if there were robust differences across the patient cohort.

Fig. 3d displays heatmaps for specific candidate m/z features that showed significant differences in abundance in OA (vs cadaveric non-arthritic) tissues. For example, the spatial distribution for the Collagen alpha-1(I) (COL1A1) peptide G^626^PAGERGEQ^634^ with m/z 900.40 (left panel) and the post-translationally modified Collagen alpha-1(III) (COL3A1) peptide G^471^EP^(ox)^GAN^(de)^GLP^(ox)^GAAGERGAP^(ox)488^ with m/z 1 626.75 (right panel) illustrated statistically significant increases of each of these peptides in the medial portions of OA tissues in comparison to the medial cadaveric non-arthritic tissues. Overall, a signature of six candidate features, including peptides from Collagen alpha-1(I), Collagen alpha-1(II), FN1, and Collagen alpha-1(VI), was significantly regulated (*P* < 0.05) comparing OA samples against cadaveric controls analyzing all four biological replicates per condition (*n* = 4 OA, *n* = 4 cadaveric non arthritic). Seven additional m/z candidate features, including peptides from Collagen alpha-1(III) and several novel features (without peptide/protein ID matches), trended towards significance (0.1< *P* < 0.05) with OA (Table S2).

Joint degeneration occurs with age and can show differential severity on one side of the joint in OA. To model and visualize disease progression with age and OA, one candidate peptide from the ROC analysis, Collagen alpha-1(I) - G^305^LP^ox^GERGRP^ox^GAP^(ox)316^ with m/z 1 211.61, was visualized and statistically quantified across both the lateral and medial halves of cadaveric ‘healthy’ donor C1 and OA patient P1 (Fig. S6A). While this Collagen alpha-1(I) feature was most highly elevated in the representative OA medial tissue (P1), the next highest intensity was observed in the medial side of the cadaveric control (C1) which was significantly higher than even in the lateral OA tissue (Fig. S6B). Therefore, MALDI-MSI detected age-related molecular OA signatures even within the cadaveric medial tissue group of ‘healthy’ controls. Even though age-related changes to OA-associated inflammatory markers and progression of articular cartilage damage markers are well known,^53,54^ here we demonstrated underlying molecular and importantly spatial changes to the subchondral bone during joint disease progression with age. These findings warrant further investigation into age-related progression of OA in larger cohorts.

### Molecular evidence of localized and site-specific OA bone damage preceding cartilage loss

**Fig. 4:**
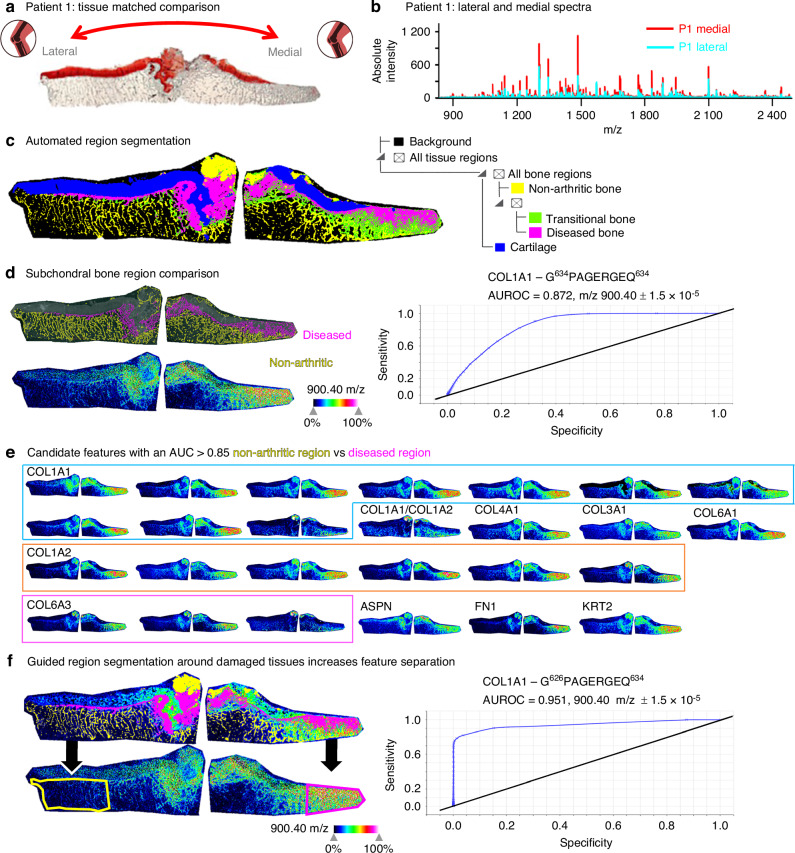
Tissue-matched comparison of OA medial vs. OA lateral tibial plateau. **a** Safranin-O staining of an OA patient (P1) showed cartilage loss in the medial joint (right) with intact lateral cartilage (left). **b** Combined MALDI-MSI spectra from medial and lateral sections revealed higher peak intensities in the medial region. **c** Hierarchical segmentation of the top 500 m/z features identified cartilage (blue) and bone subtypes: healthy trabecular bone (yellow), transitional layer (green), and diseased bone beneath cartilage loss (magenta sclerotic bone). **d** Pairwise comparison of non-arthritic vs. diseased bone regions identified features with AUROC ≥ 0.85. A representative Col1a1 peptide (G^626^PAGERGEQ^634^, m/z 900.40) localized to diseased bone had a predicative AUROC value of 0.872 using only automated segmentation protocols. **e** In patient P1, 34 discriminatory features were identified between non-arthritic and diseased bone. **f** Manual segmentation of the same molecular data was completed to demonstrate that segmentation guided by both automated hierarchical segmentation and physiologic insights produced improved AUROC performance. This novel manual segmentation method outlining the diseased subchondral bone specifically and only below the area of cartilage loss (magenta outline) vs. the healthy intact lateral bone (yellow outline) increased AUROC performance from 0.872 (**d**) to 0.95 for the representative Col1a1 peptide at m/z 900.40

Given the small sample size, the heterogeneity inherent to human patient samples, the many distinct endotypes of OA,^55,56^ and natural age-related damage in the non-arthritic cadaveric joint cohort, analysis utilizing the lateral half of the OA joints as a patient matched control was justified to further investigate the molecular markers of OA found by MALDI-MSI (Fig. 4a). Independent data sets for both halves of the tibial plateau from representative OA patient P1 were loaded into the same SCiLS data processing workspace to begin this new analysis following the same ‘candidate-validation’ approach used in the previous comparison. A composite spectrum was generated that represents the combined 64 183 individual spectra across the entire tibial plateau of OA patient P1 (Fig. 4b). Region segmentation (Fig. 4c) recapitulated similar bone regions found in the Medial OA section as before, including the non-arthritic medial subchondral bone (yellow) and a progressive change towards the most damage diseased bone region on the most medial edge (magenta). Interestingly, this diseased bone signature extended well into the lateral side of the joint where a thick cartilage layer remained and is not limited to the area of cartilage loss on the medial edge. The segmented regions containing the diseased bone signature and the healthier bone signature were separated out and a discriminating feature analysis utilizing the ROC prediction method was performed to generate candidate features with an AUROC > 0.85. A heat map for the example candidate peptide Collagen alpha-1(I) - G^626^PAGERGEQ^634^, with m/z at 900.40 and an AUC of 0.872, is shown beside its ROC curve to illustrate how well the distribution of the candidate features from this analysis aligns to the boundaries of the segmented regions (Fig. 4d). Overall, 34 candidate features with an AUC > 0.85 (Table S2), each with their spatial distributions, were found in this comparison to generate candidate molecular features for later cohort “validation” (Fig. 4e). To verify that the candidate lists were not overly sample-specific, this same candidate feature identification process was completed on an additional OA patient (Fig. S7), and this resulted in a similar number of candidate marker features with the majority of features in both lists.

Since the “diseased” region segment (magenta) that contains the diseased tissue appears somewhat underneath lateral cartilage and is not constrained solely to the medial tissue, we restricted comparisons to just the outermost regions of the medial and lateral joint to discriminate joint-site specific differences between diseased and healthier subchondral bone from the same donor (Fig. 4f). New ROIs were generated using both histology and the automatically generated region segments as guides. Despite these new regions now containing multiple tissue types (marrow, trabecular bone) the predictive AUROC for many of the candidate features, including the depicted bone marker Collagen alpha-1(I) peptide, increased with the guided segmentation.

**Fig. 5:**
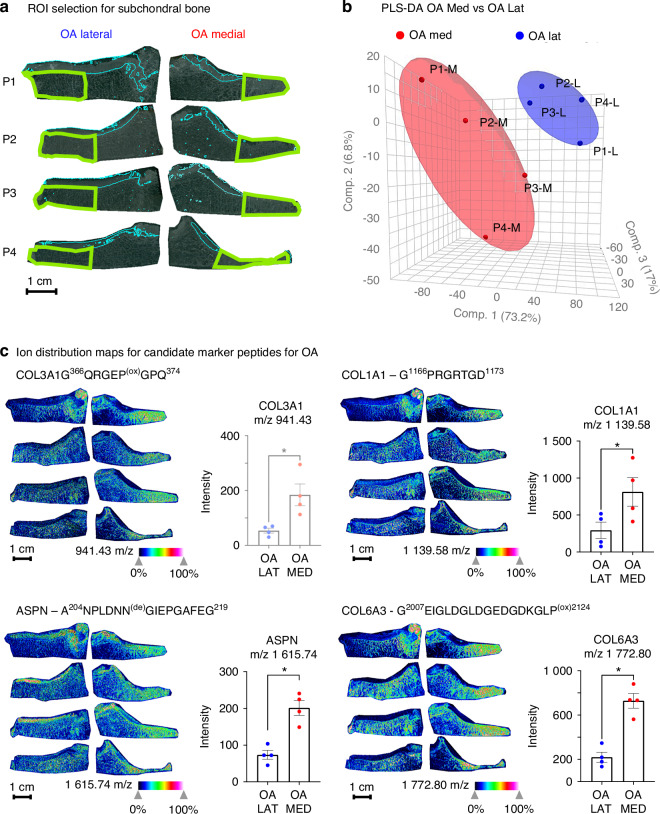
Candidate Ion validation Across Multiple OA Patients Comparing Medial to Lateral Joints. **a** Regions of Interest (ROIs in green) were manually segmented around the outer most regions of the medial and lateral tibial plateaus of OA patients (*n* = 4), using the edge of the cartilage layer (thin blue line) as a guide for affected tissue in medial joints and using an equivalent distance on the lateral side. Average pixel intensity within the segmented region was calculated for each candidate ion that was previously identified using automated tissue segmentation and ROC curve predictive score. **b** Visualization of data grouping by 3-dimensional PLS-DA of pixel intensity showed distinct separation between the two joint sides despite physiologic complexity and patient heterogeneity in the OA medial cohort. **c** Group-wide heat maps of candidate feature distribution with peptide IDs derived from several ECM proteins or related proteins, Collagen alpha-1(III) (Col3A1) G^366^QRGEP^(ox)^GPQ^374^ at m/z 941.43, Collagen alpha-1(I) (Col1A1) G^1166^PRGRTGD^1173^ at 1 139.58 m/z, Asporin (ASPN) A^204^NPLDNN^(de)^GIEPGAFEG^219^ at m/z 1 615.74, and Collagen alpha-3(VI) (Col6A3) G^2007^EIGLDGLDGEDGDKGLP^(ox)2124^ at m/z 1 772.80, depicted increases of the ion intensity in the outermost region of medial OA joints compared to their lateral counterparts (of the same OA patient). Statistical differences in feature intensities were confirmed via paired *t*-test *P < 0.05 (*n* = 4) *see* Table S2

Finally, upon combination of both the candidate features from the initial comparisons of control cadaveric medial tissue to OA medial tissues (described in Fig. 3) and the comparisons between OA lateral to and OA medial tissues (described in Fig. 4), we obtained 44 unique candidate features or peptides that we subsequently assessed and ‘validated’ in the complete OA and cadaveric cohort (*n* = 4 for each group). Guided segmentation was completed for the outer most edges of lateral and medial portions of each OA tibial plateau (Fig. 5a). A PLS-DA based on the mean pixel intensity of all 44 candidate features from each tissue section (Fig. 5b) showed that OA lateral samples cluster away from the OA medial samples despite them originating from the same donor. Next, individual candidate features were “validated” by the mean pixel intensity for the candidate across the 8 tissue sections (4 OA medial vs 4 OA lateral) with a paired *t*-test. Utilizing all 44 features for these comparisons gave a more comprehensive assessment of the molecular regulation occurring in OA. For instance, the candidate Collagen alpha-1(I) peptide G^305^LP^(ox)^GERGRP^(ox)^GAP^(ox)316^ with m/z 1 211.60, initially only identified by comparing OA medial P1 to cadaveric medial C1, did not show significance on the cohort level in the earlier comparison of medial control cadaveric tissues to site-matched medial OA tissues (*P* = 0.229), but did show significant elevation in the OA cohort when comparing OA Medial to OA Lateral (*P* = 0.031) (Table S2).

In statistical comparisons of the OA medial tissues vs their lateral tissue-matched controls using a paired t-test, 30 of the 44 candidate features showed significant differences, while an additional 8 features trended towards significance (Fig. S8). Of the 44 candidates, 36 m/z features matched to peptide identifications within 2 mDa of their theoretical mass from previous studies and our own spectral libraries generated^20,43,48^ (Table S2). Of the statistically elevated candidates in OA medial vs lateral tissues with known peptide identifications, four peptides belonging the fibrillar collagens Collagen alpha-1(III) and Collagen alpha-1(I), the network forming Collagen alpha-1(VI), and a component of the cartilage ECM, Asporin are depicted (Fig. 5c). Of the eight unidentified candidates, five reached significance levels in the group-wise comparison suggesting these may be novel bone-specific markers of OA that will require further classification, representing exciting possibilities for future worked concerned with subchondral bone degeneration in OA. Quantification of the full 44 candidates, with the additional 13 candidate features identified from this OA medial to OA lateral comparison, showed further differences in the earlier OA medial to control cadaveric medial comparison (Fig. S9). Although ASPN was the only non-collagenous protein that was discovered as significantly elevated in OA in this study, other non-collagenous proteins, including MMPs and structural cartilage proteins such as Chondrohaderin, were detected and identified (Fig. S10). Overall, we demonstrate the capabilities of MALDI-MSI and an ECM-specific collagenase-based enzymatic digestion to detect osteoarthritis progression in the subchondral bone of human tibial plateaus and the potential to identify novel markers of disease (Fig. S11).

### Spatial distribution of hydroxyproline post-translational modifications – enrichment of collagen crosslinking in OA subchondral bone

**Fig. 6:**
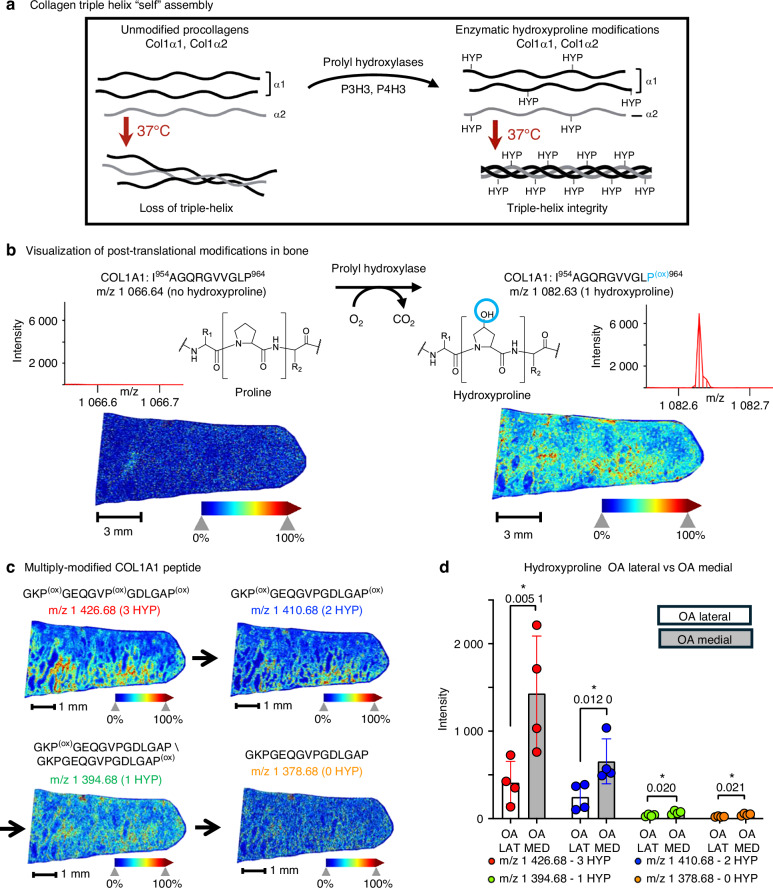
Post-translational modifications of collagen in OA subchondral bone. **a** Proper assembly of procollagens into the tropocollagen triple helix requires post-translational modifications such as prolyl hydroxylation. **b** An unmodified Col1A1 peptide (I^954^AGQRGVVGLP^964^, m/z 1 066.64) was nearly undetectable in OA subchondral bone, whereas the hydroxyproline-modified form (I^954^AGQRGVVGLP^(ox)964^, m/z 1 082.64) showed strong trabecular localization. **c** A Col1A1 peptide with three hydroxyproline residues (G^656^KP^(ox)^GEQGVP^(ox)^GDLGAP^(ox)670^, m/z 1 426.68) displayed high signal, while partially modified forms with 2, 1, or 0 HYP showed progressively weaker intensity and lower spatial definition. **d** Quantification across replicates (*n* = 4) from medial (grey) and lateral (white) OA tibial plateaus confirmed significant differences in hydroxyproline-modified peptide abundance (*P < 0.05, paired *t*-test)

One important mechanism in bone development and remodeling is the tightly regulated series of post-translational modifications that are required for the self-assembly of collagen fibrils (Fig. 6a). This self-assembly requires the oxidation of specific proline residues along different collagen subtypes, which is catalyzed by several enzymes, including the family of prolyl hydroxylases.^57,58^ These homeostatic mechanisms are tightly regulated in healthy bone and joint tissues but have been implicated as dysregulated in different models of skeletal disease and aging in mice, including in diabetes and in disrupted osteocytic perilacunar/canalicular remodeling.^14,15^ Thus, the ability to detect specific post-translational modifications on distinct amino acids within a protein sequence from skeletal tissue, and the determination of their quantitative differences during disease or aging, will be a helpful tool to assess the molecular alterations occurring in aging and disease.

MALDI-MSI along with peptide spectral libraries from collagenase III digested tissues demonstrated the ability to detect and visualize matching peptides containing one or more oxidized proline residues in bone tissues. One peptide feature at m/z 1 082.63 displayed very high signal intensity in the subchondral region below areas of cartilage loss in an OA patient (Fig. 6b, right). This feature matched to a peptide from Collagen alpha-1(I) with a hydroxyproline (HYP) modification, I^954^AGQRGVVGLP^(ox)964^, with a small mass error ( < 1.5 × 10^−5^). We also visualized the spatial distribution of the corresponding peptide without HYP modification (unmodified proline) I^954^AGQRGVVGLP^964^ with a precursor ion at m/z 1 066.64 (Fig. 6b, left). The latter unmodified peptide was not detected above noise level and did not generate any image with discernable bone trabecular morphology, while the HYP modified peptide was highly abundant. To confirm the identity of the modified HYP peptide we performed liquid chromatography tandem mass spectrometry (LC-MS/MS) data-dependent acquisitions (DDA) using the timsTOF HT (Bruker) of collagenase digested tissues. This confirmed the identity of the modified peptide and the specific site-location of the modified proline, Pro^964^ in the peptide IAGQRGVVGLP^(ox)^ of Collagen alpha-1(I) on the MS/MS level (Fig. S12A). A precursor for the unmodified peptide, I^954^AGQRGVVGLP^964^, was not detected in this LC-MS/MS experiment consistent with the MALDI MSI data (described above). The presence of the modified peptide I^954^AGQRGVVGLP^(ox)964^ and the absence of the corresponding unmodified form emphasized a dramatic shift in proline modifications during OA and the potential of these peptides to discriminate mature or diseased skeletal tissues.

This integrated workflow of combining MALDI-MS Imaging and ESI-MS/MS spectral library building was used to determine which modified forms of peptides - specifically hydroxy proline-containing peptides - were more abundant with disease or OA. For example, another feature at m/z 1 426.68 displayed an abundant spatial profile with localized areas of high intensity near the center of the bone trabecula in a region of sclerotic OA subchondral bone (Fig. 6c). This feature (m/z 1 426.68) matched to a triply-modified peptide of Collagen alpha-1(I), G^656^KP^(ox)^GEQGVP^(ox)^GDLGAP^(ox)670^. A feature at m/z 1 410.68, corresponding to the same peptide sequence with one less HYP, G^656^KP^(ox)^GEQGVPGDLGAP^(ox)670,^ presented with a similar spatial distribution including similar regions of signal intensity within the trabecular bone. LC-MS/MS DDA confirmed the presence of peptide precursors in our data set with three (Fig. S12B) and two HYP residues (Fig. S12C) for this peptide. MS/MS spectra for the doubly HYP modified peptide were able to distinctly localize the position of the two HYP modifications to prolines Pro^658^ and Pro^670^.

Attempts to assay this peptides sequence with yet fewer post-translational modifications, such as the same base peptide with 1 HYP (at m/z 1 394.68), and the unmodified peptide (at m/z 1 378.68), resulted in low abundant signals in the MALDI MSI spectra (high signal to noise). Additionally, precursor ions for these species were not detected in the corresponding LC-MS/MS acquisitions and the generated spectral library. Comparisons investigating the OA cohort (Fig. 6d) for this Collagen alpha-1(I) peptide in all its HYP-modified forms, starting with the triply modified form G^656^KP^(ox)^GEQGVP^(ox)^GDLGAP^(ox)670^, with m/z at 1 426.68 and the doubly modified form at m/z 1 410.68 (2x HYP) were highly abundant and significantly upregulated in the diseased OA tissue (OA medial- grey bar vs OA lateral – white bar). We also extracted pixel intensity for corresponding peptides with only 1 HYP at m/z 1 394.68 or 0 HYP at m/z 1 378.68. However, these average pixel intensities were extremely low, not reaching above the surrounding signal-to-noise (S/N) background, and they were observed at almost 30 times lower intensity than the triply-modified peptide. Overall, we determined that collagen-derived peptides containing (multiple) HYP modifications were more prevalent in bone material and - more importantly - can become over-represented in OA and disease tissues vs control (healthy) tissues.

**Fig. 7:**
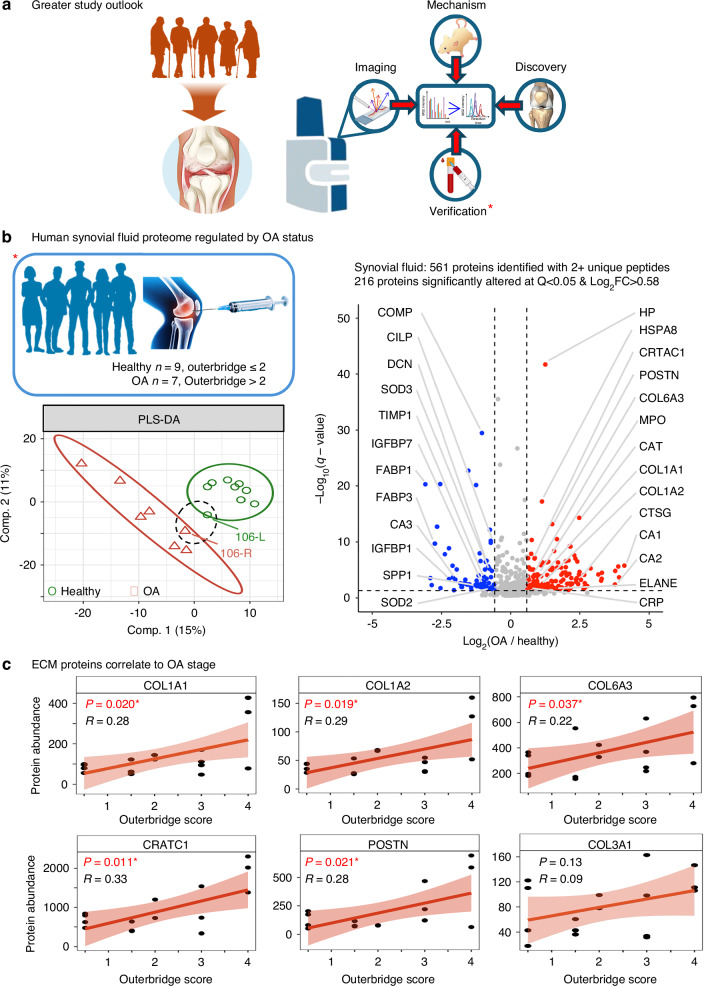
Integration of Spatial MALDI-MSI, Synovial Fluid Proteomics, and Mouse Models for OA Biomarker Discovery. **a** Spatial mass spectrometry imaging (MALDI-MSI) of OA knee joints identified candidate proteins for mechanistic and biomarker studies. Future work will expand to larger TKA cohorts, including sex-specific analyses, with validation in vitro, in vivo, and in OA mouse models. **b** Synovial fluid proteomics (DIA-MS) from knees of OA patients (red triangles, Outerbridge >2) and controls (green circles, Outerbridge ≤2) revealed distinct clustering by PLS-DA. Donor 106 showed inter-knee severity differences. Differential analysis identified 216 significantly altered proteins (Q < 0.05, Log_2_FC > 0.58), including COL1A1 and COL6A3, also upregulated in OA subchondral bone by MALDI-MSI. **c** Regression analysis highlighted several ECM and known OA proteins (CRTAC1, POSTN) that significantly correlated with OA stage and found several markers that were consistently regulated in synovial fluid (DIA-MS) and subchondral bone (MALDI-MSI) (COL1A1, COL1A2, COL6A3)

In order to independently validate identified peptide and protein candidates detected by MALDI-MSI in this relatively small prospective cohort, we collected synovial fluid from the joints of a separate cohort of human donors for global proteomic analysis with Liquid Chromatography Data-Independent Acquisition (DIA) Mass Spectrometry (LC-MS/MS, DIA-MS, Fig. S13). The paired analysis of this separate human cohort also demonstrated the utilization of MALDI-MSI as a part of a larger comprehensive workflow including biofluids, e.g. synovial fluid (Fig. 7a). This additional cohort was composed of synovial fluid from the right and left joints of middle to late aged individuals. Each joint was independently scored for OA stage using the Outerbridge scoring system^59^ (Table S4). The DIA-MS workflow quantified 561 proteins from human synovial fluid uncovering a broad functional array for proteins involved in immune response, proteolysis, antioxidant response, protein binding, and bone-specific markers (Fig. S14A–C, Table S5A). Partial Least Squares Discriminate Analysis (PLS-DA) for the obtained synovial fluid proteomics data demonstrated that the individual knees clustered distinctly by Outerbridge score, clearly defining a Healthy group (Outerbridge ≤2) and an OA group (Outerbridge >2). In addition (and as expected), synovial fluid from knees obtained from the same individual donor clustered closely together. One donor presented with differing Outerbridge scores for each knee (Donor ID: 106) splitting up into the Healthy and OA groups (Fig. 7b). Differential analysis comparing synovial fluid from OA knees to synovial fluid from healthy knees determined that 216 proteins were significantly regulated between the two groups at Q < 0.05 & Log_2_FC > 0.58 (Table S5B). This included upregulation of the known OA marker Cartilage acidic protein 1 (CTRAC1) in the OA group as well as markers that we also identified as upregulated in OA subchondral bone by MALDI-MSI, such as COL1A1, COL1A2, and COL6A3, among others. Downregulated proteins interestingly included several markers for cartilage ECM including COMP, CILP, and DCN. Overall, we observed a large functional shift for synovial fluid proteins in OA patients revealing upregulation for proteolytic and catabolic pathways and downregulation for several proteins with metabolic functions (Fig. S14D). Importantly, in the synovial fluid several ECM proteins showed significant positive correlation with increasing OA stage by Outerbridge score including candidates that we found by MALDI-MSI (see above), such as COL1A1, COL1A2, and COL6A3 and other recognized markers for OA or inflammation, including CRTAC1 and POSTN (Fig. 7c). Lastly, COL3A1, that was found significantly upregulated in OA subchondral bone via MALDI-MSI, showed a trend (however non-significant) towards correlation with OA grade (Fig. 7c). The proteins mentioned, are of key interest for future human OA studies interrogating synovial fluid as potential marker proteins.

## Discussion

Osteoarthritis is most commonly first indicated by self-reported pain in patients but also radiographically by the appearance of osteophytes, or mineralized growths, and by joint-space narrowing seen on X-rays or Magnetic Resonance Imaging (MRI) scans. Even then, full confirmation or grading of the disease cannot be made until histopathology or visualization of the exposed joint can confirm the loss of cartilage and other markers of disease. Further, there are currently only limited pre-clinical molecular diagnostics for OA or OA progression that stratify patients who may benefit from early intervention to improve joint health. In this study we demonstrate that matrix-assisted laser/desorption ionization (MALDI) mass spectrometry imaging (MSI) may be used as an effective tool for the study of the molecular and biochemical spatial changes that occur in human joint tissues during osteoarthritis (OA) and that molecular insight gained from these studies can inform development of non-invasive diagnostic biomarkers for disease progression.

We report the first successful protein-based molecular spatial mapping of skeletal tissues composed of both cartilage and bone regions by targeting ECM proteins with an enzymatic strategy utilizing collagenase-III. The feature-rich composite spectrum of collagenase III digested skeletal tissue captured by MALDI-MSI molecularly discriminates bone from cartilage, demonstrating the effectiveness and promise of this technology to study musculoskeletal disease. Proteins detected comprise standard ‘marker’ proteins for bone and cartilage, including Collagen alpha-1(I) and Collagen alpha-1(II) respectively, spatially localized to their corresponding tissue types as confirmed by histology. Additionally, higher resolution imaging at the border between bone and cartilage identifies specific molecular features with spatial distributions that can recapitulate features found through traditional histopathologic approaches. Molecular image processing allowed for parallel assaying of over several hundred candidate peptide features at once, enabling a comprehensive workflow for unbiased detection of novel disease markers. In FFPE tissue sections from the tibial plateau of total knee replacement patients and site-matched tissues from non-arthritic, age, and BMI matched cadaveric donors, we find specific peptides belonging to ECM proteins that are upregulated in OA. These include peptides belonging to less common collagens such as Collagen alpha-1(III) and Collagen alpha-3(VI). The identified panel of peptide markers was expanded by utilizing the less affected lateral half of joints from the same OA donors as patient and tissue matched controls. This limited person-to-person variability and allowed for the validation of over 30 specific peptides that were significantly upregulated in medial OA tissues compared to either cadaveric medial or OA lateral controls. Interestingly, in our analysis, no candidate features were significantly down regulated in OA subchondral bone among our small clinical cohort. This could be an effect of the small cohort size or the targeted use of collagenase III to investigate matrix protein changes, or our primary focus on bone markers in end-stage disease conditions. Still, the identification of protein level regulation in the subchondral bone of OA suggests that molecular remodeling on the protein level occurs throughout the process of joint degeneration in addition to alterations to bone density and bone mass. Knowledge of how different peptide regions of ECM proteins are altered in disease provides needed biologic context for the mechanical and biochemical changes occurring in OA diseased tissues that may directly influence resident cell types. Additionally, the presence of critical candidate peptides in the synovial fluid of separate OA patients motivates the use of these peptides as therapeutic or diagnostic biomarkers in future studies. This study provides the successful application of MALDI-MSI for protein-based imaging on composite human joint tissues including both cartilage and bone, as well as biologic insight into joint disease on the protein and molecular level which can inform future studies aimed at improving treatment and diagnostics for OA.

A significant insight offered by this OA imaging study is the direct visualization of the spatial distribution of ‘diseased’ subchondral bone on the protein level. While it is intuitive that damage and associated changes to the ECM would be most highly constrained to areas of subchondral bone underneath lost cartilage, proteomic profiling of altered bone ECM composition has not before been achieved. These changes primarily occur in areas where osteocyte dysfunction and subchondral bone sclerosis have been described histologically and radiographically (Fig. S1), as demonstrated in previous studies on this cohort of patients.^7^ While this disease signature is most evident in areas underlying cartilage loss, imaging of the lateral half of OA joints reveals that the disease signature emerges even in subchondral bone areas where a thick cartilage layer remains (Fig. 4c purple, Fig. 5c). While osteocyte dysfunction plays a causal role in OA, alterations to subchondral bone also result from perturbations to MSC, osteoblast and osteoclast function; thus it will be important to further investigate the cellular origins of this altered proteomic signature.^7,60^ Such studies may elucidate the extent to which progression of OA arises from altered joint mechanical loading, changes in cellular metabolic or secretory behavior, or altered signaling by resident skeletal or immune cell populations. Since the loss of subchondral bone homeostasis can precede cartilage damage, the search for early OA diagnostics should focus on bone. Thus, the continued investigation into subchondral bone changes, especially at earlier ages, among a larger cohort of individuals is warranted. Additionally, given that the current OA cohort was limited to male patients enrolled in the Veteran’s Administration healthcare system, and that OA has an increased prevalence in females,^1,2^ proteomic profiling of OA in female joints is essential and should be prioritized in future studies.

Of the many exciting findings from the molecular imaging of subchondral bone in osteoarthritis, one major outcome was the identification of multiple collagen species within the disease signature of subchondral bone. Here, an enzymatic pretreatment of the tissues was completed with collagenase III to target the ECM and ECM-associated proteins. Due to the nature of collagenase III, it is not surprising that many members of the candidate features identified in this study are a form of collagen, although many other non-collagen ECM proteins were detected by this analysis (Fig. S10). Collagenase III, or MMP13, is already known to be active and contribute to OA related damage,^7,61^ and so additional application of this enzyme to the tissue may have masked potential endogenous or existing damage marker peptides. However, given that the dramatic increase in signal for damage marker peptides is localized to sclerotic regions of subchondral bone under cartilage loss and not in areas of remaining subchondral bone exposed to the same enzymatic pretreatment process, this enzymatic processing accurately allowed for the identification of broad molecular changes occurring in subchondral bone with OA. Additionally, the use of FFPE tissues that were exposed to thorough solvent washing and paraffin infiltration may have removed unbound, endogenous marker peptides.

Here, we provide molecular evidence of bone remodeling in OA on the protein level through the regulation of several collagen subtypes in bone. The regulation of these specific collagens and ECM proteins in disease can additionally give insight into biologic mechanisms of disease. Collagen itself is not simply a structural protein, for example it is well known that the collagen fibril is bioactive and can bind many other proteins or provide Arginine-Glycine-Aspartic (RGD) domains for cellular attachments.^62–64^ In a specific example, the unmodified Collagen alpha-1(I) peptide G^1165^PRGRTGD^1173^ detected at m/z 1 139.58 is significantly increased in the diseased medial regions of our OA patients (Fig. 5c). This specific peptide exists towards the C-terminal domain of the collagen 1 triple helix and lies within an area of interleukin 2 (IL2) and amyloid precursor protein (APP) binding^63,64^ (Fig. S15A). IL2 is a well-known inflammatory marker and enhancer of the T-cell immune response, while APP interaction with collagen 1 has also been shown to enhance cell-matrix adhesion and enhance the function and activation of peripheral monocytes.^65,66^ Thus, the detection of this specific peptide in the subchondral bone of OA can point towards molecular mechanisms by which inflammation may be amplified in OA. This peptide also overlaps with an area of Phosphophoryn, also known as Dentin phosphoprotein - a protein known to enhance mineralization in the formation of dentin tissue.^67^ The implication of proteins involved in mineral regulation in OA-affected tissue could provide mechanistic insight into the deregulation of mineralization and subchondral bone thickness that occur in OA, though these interactions remain to be investigated. These novel observations in the context of OA and knowledge of protein spatial distribution specifically in the subchondral bone beneath areas of cartilage loss provide new insights into the cellular and molecular interactions that may be occurring in disease and promote further investigation of the biologically regulatory features of ‘structural’ collagen in the context of this disease.

Other collagen I peptides identified as upregulated in OA when compared against non-arthritic, cadaveric controls or histologically normal lateral joint tissues, are those identified with post-translational modifications. Hydroxyproline modifications in bone are important enzymatically generated modifications that increase collagen structural integrity and reinforce the triple helix of the collagen fibril.^58,68^ The upregulation of these modifications in OA suggests molecular mechanisms impacting the material stiffness of bone in OA joints independently of hypermineralization. Further examples of PTM regulation in OA can be found in other upregulated regions of the collagen type 1 fibril. For example, the peptide of Collagen alpha-1(I) G^626^PAGERGEQ^634^ at m/z 900.40 within the mature collagen type I fibril is bound to a region of Collagen alpha-1(II) that contains lysine Lys^343^ - a known glycation site (Fig. S15B).^64^ This region of the mature collagen fibril is also thought to bind different keratin sulfate proteoglycans (Fig. S15B), both important PTMs for collagen.^63,64^ Another peptide directly identified with HYP modifications and upregulated in OA, G^565^KPGEQGVPGDLGAP^670^ also contains a documented glycation site at Lys^657.^ Glycation, in the form of advanced glycation end-products (AGEs), can further stiffen the bone matrix^69^ while alterations to proteoglycans in bone, such as in diseases like osteogenesis imperfecta, negatively impact bone strength.^70^ 22 of the 27 significantly elevated candidate features identified as belonging to Collagen alpha-1(I) or Collagen alpha-1(II) contained glycation or other known locations of collagen post-translational modifications that are not ascribed to HYPs when compared to the functional collagen interactome (Table S2). Thus, the presence of these modification sites in OA bone where cartilage has worn away implicates post-translational regulation as one mechanism that may alter subchondral bone homeostasis in OA. Interestingly, we find that these PTM changes occur in areas of cartilage loss where it is thought that physical loading of the joint alters throughout the course of disease.^71^ It remains to be elucidated whether these changes reflect the mechanical reinforcement of collagen by PTMs in bone underlying areas of cartilage damage or are a mark of underlying disease in bone that gives rise to local cartilage degeneration.

Aside from collagen 1, the presence of other less common collagens comprises the detected disease signature of OA related peptides in bone. Several peptides of Collagen alpha-1(III), a less common fibrillar collagen active in wound repair, is upregulated in the diseased subchondral bone of OA patients (Fig. 5c). Collagen Type III has also been implicated in the cartilaginous regions of osteoarthritic joints as an attempt by chondrocytes to remodel or stabilize remaining cartilage,^72^ while in bone it is thought to promote osteoblast differentiation leading to increases in trabecular bone mass.^73^ Thus, the upregulation of Collagen Type III in the subchondral bone of OA tissue compared to control tissue points to a potential role in driving late stage OA increases to bone mass and trabecular bone sclerosis common in OA.^74^

Another less common collagen type detected in OA subchondral bone through MALDI-MSI was Collagen Type VI. Though commonly seen in muscle and tendon, in the pericellular matrix around chondrocytes,^75^ and in young actively remodeling bone,^76^ this, to our knowledge, is the first documented detection of Collagen Type VI in aged-human arthritic bone. This adds to a growing base of knowledge of the role of Collagen Type VI as a fibrotic biomarker outside its role in tissue development,^77^ now specifically in OA. The detection of various peptides of Collagen alpha-1(VI) and Collagen alpha-3(VI) (Fig. 5c, Table S2), may be the first detection of this network forming collagen in adult bone tissue outside the growth plate where chondrocytes aid in bone formation through endochondral ossification.^78,79^ Knockout of the *Col6a2* gene prevented normal ossification of the temporomandibular joint in mice, suggesting that Collagen Type VI may play important roles in chondrocyte differentiation and bone growth in development,^78^ however its role in *bone* in OA is unknown. Collagen Type VI is produced by hypertrophic chondrocytes in arthritic cartilage where it can modulate pericellular mechanics.^75,80,81^ Strikingly, our detection of Collagen Type VI in OA subchondral bone hints at an important role for this collagen subtype in OA related bone remodeling and fibrosis. Although precise mechanisms behind this and the cell type responsible for its presence in osteoarthritic bone are currently unknown, the role of Collagen Type VI during bone development implies this collagen subtype may mark the effort to mount a regenerative response, which may ultimately contribute to subchondral bone sclerosis.

The presence and upregulation of multiple collagens in the synovial fluid of a separate cohort of OA patients analyzed orthogonally by global LC-MS/MS further confirmed our findings of these proteins (and their relevance during OA) by our spatial MALDI-MSI in subchondral bone of the knee joints. We hypothesize that subchondral bone remodeling in OA resulted in the release of factors from the sclerotic and inflamed knee into the synovial space. While COL1A1 and COL1A2 were upregulated in the synovial fluid from OA knees (and these presented as strong candidates by the spatial MALDI-MSI also), the similar upregulation of the less common collagens COL6A3 and COL3A1 in the synovial fluid with OA is more exciting in that collagens 3 and 6 are non-homeostatic collagens for bone tissue. Collagens 3 and 6 are typically more abundant in connective tissues in healthy conditions and are only seen in low amounts in our healthy synovial fluid extracts. While the appearance in OA synovial fluid may result from inflamed synovial lining tissues, the direct detection of these collagens in OA subchondral bone via MALDI-MSI suggests a stronger relationship to bone remodeling in OA. Interestingly, structural markers for cartilage were found downregulated in OA synovial fluid, including COMP, CILP, and DCN, while Collagen II was not detected. This also is in line with our MALDI-MSI results in that we found no significant changes to Collagen II in cartilage while other cartilage matrix proteins were infrequently detected. This supports the conclusion that a focus on subchondral bone remodeling and its disease signature will be a tractable route toward diagnostic development rather than a focus on cartilage markers. While the peptide and protein disease signatures for both subchondral bone and synovial fluid contain many different collagen peptides, the specific peptide and protein identities and their functional and biological roles offer insight and motivate further studies into the cellular and molecular mechanisms at play in subchondral bone in OA.

### Conclusions and future outlook

Overall, this study presents the first use of MALDI-MSI to investigate skeletal proteins in human joint disease providing further molecular knowledge about the progression of joint disease in subchondral bone. We show that markers of joint disease and OA are detectable in the subchondral bone, that these may be evident prior to cartilage loss, and that novel, specific regions of common ECM proteins may be hallmarks of this damage. Additionally, we demonstrate that key proteins found in sclerotic subchondral bone are significantly upregulated in the synovial fluid of OA patients as well.

Thus, by defining the spatial molecular landscape of OA on the protein level, this study advances new opportunities to increase the specificity of disease diagnostics and biomarkers for OA and opens new opportunities for future works with potential to establish new modes of disease tracking in human osteoarthritis. Further studies that implement MALDI-MSI to investigate the progression of human OA may be completed to extend the impact of this technological advance. First, there remain mainly promising molecular features and candidate peptides upregulated in our OA cohort that currently do not have matching IDs. In future work we will pursue these features by expanding existing skeletal tissue ECM specific libraries for the identification of novel biomarkers of disease. Additionally, emerging technologies are allowing in situ MS/MS (MS2) during MALDI-MSI that can accurately identify analytes during imaging experiments without the need to remove and digest imaged tissue. This will increase the ability to directly confirm protein IDs in place during imaging experiments, circumventing longer workflows utilizing LC-MS/MS for library building. Still, the promising success of this collagenase-based imaging motivates the use for other serial enzymatic digestions including elastases and chondroitinases^46^ that can further probe the compositional changes of the ECM in OA. Excitingly, our new molecular evidence supports previous morphologic findings from microCT and MRI that subchondral bone in OA undergoes dramatic molecular remodeling, and these changes to subchondral bone can occur in bone even before cartilage loss in some cases.^5,6,10^ Thus, we suggest continued effort be spent in understanding the changes to subchondral bone as a way to develop diagnostics and treatments for osteoarthritis.

Osteoarthritis is a complex, personalized disease with multiple etiologies. OA endotypes exist that stem from conditions such as traumatic injury (post-traumatic OA), obesity, and even normal healthy aging, among many others, imparting a natural variability to this disease. In this prospective proof-of-concept study, to validate the detection of novel peptide markers in OA subchondral bone, we analyzed a separate cohort of patients in a distinct biologic matrix, namely synovial fluid. This independent analysis demonstrated how utilizing MALDI-MSI in conjunction with orthogonal analyses, HPLC DIA-MS/MS, on a separate human cohort could model future studies enrolling larger cohorts of individuals with OA. This is significant in that OA disease burden is skewed such that cases of total knee replacement for woman are more than 33% higher than for men,^82^ while our limited cohorts are biased towards male donors from the VA system. Thus, we suggest future studies utilizing MALDI-MSI and HPLC DIA-MS/MS to include both male and female patients, at multiple disease stages and ages as a part of a larger comprehensive study as a novel way to interrogate joint remodeling (Fig. 7a). Additionally, future screens of larger human populations with spatial MALDI-MSI can utilize tissue micro arrays (TMAs) to increase throughput and analyze as many as 60 individuals on one slide,^39^ thus matching throughput capabilities of HPLC MS/MS approaches. The ability to visually detect and validate diagnostic biomarkers across a range of patients and samples would advance avenues of personalized medicine in the orthopedic field and in the context of OA. Thus, by identifying and tracking endogenous markers that increase with OA in subchondral bone, and matching them to disease signatures in biofluids (e.g., synovial fluid), we can see a path towards the development of highly sensitive and specific diagnostics for a disease that currently lacks reliable early detection methods. Additionally, this technology may be effectively utilized in other arthritic and orthopedic disorders including rheumatoid arthritis, ectopic mineralization, and spondylarthritis to further advance research into these elusive conditions.

The extension of this technology to mouse models of OA where the biologic mechanism may be more precisely traced, and the disease progression and OA stage controlled, would provide much needed time course data and new hallmarks of early disease and tissue remodeling (Fig. 7a). Classification of the protein changes occurring in subchondral bone early in disease, prior to cartilage loss or the clinical presentation of pain, would greatly enhance the ability to develop diagnostics. In addition, knowledge gained from foundational mass spectrometry studies, such as this, can be used to identify precise molecular candidates from murine studies and larger panels of candidate peptides can be screened in biofluids from larger, healthy human populations (Fig. 7a). This circumvents the inherent difficulty and limitations of analyzing skeletal joint tissues from human patients with end-stage OA.

MALDI-MSI paired with targeted ECM enzymatic treatment has proven to be an effective tool to interrogate the molecular changes occurring in human joint tissue. We have demonstrated its potential for use in both discovery and potentially diagnostic-based workflows, providing new much-needed tools to investigate skeletal biology in a spatial manner. Findings from our work will motivate the use of spatial MALDI-MSI and new technologies to further provide novel protein-based biomarkers for OA. The combination of MALDI-MSI results with powerful proteomic analyses of synovial fluid from human OA patients provided candidate validation and opens opportunities for many future applications in the context of translational biomarker research.

## Materials and methods

### Human donor population and knee joint specimen preparation

Human knee joint tissues were obtained as described from the University of California, San Francisco, UCSF, as pre-prepared formalin-fixed paraffin-embedded (FFPE) tissue sections from an existing tissue bank.^7^ Briefly, four tibial plateaus from a group of non-OA diagnosed donors (cadaveric controls C1-C4) without a history of osteonecrosis, osteoporosis, or fractures were collected through the Willed Body Program at the University of California, San Francisco, and four osteoarthritic tibial plateaus were collected as surgical discard from patients receiving total knee arthroplasty/replacement, with an age range from 64–79 years old, (Patients P1-P4), who were clinically diagnosed with stage IV Osteoarthritis of the medial tibial plateau of the knee (Figs. S1 and 2). Recruitment occurred through referral from orthopedic surgeons at the Department of Veterans Affairs Medical Center (San Francisco, CA, USA). This resulted in an age, sex, and BMI matched cohort of human tibial plateaus that were stratified by their Osteoarthritis Research Society International (OARSI) score as graded by clinical pathologists as determined from histopathologic Safranin-O/Fast Green stains (Figs. S1 and 2, Table S1). All samples were collected with informed consent from patients with OA of the femorotibial joint as described in protocols that were reviewed and approved by the Human Subjects Protection Program Institutional Review Board of UCSF and the Department of Veterans Affairs Medical Center.^7^ All samples were harvested within 4 days postmortem to minimize the effects of degradation. Tibial plateaus, composed of the articular cartilage and underlying subchondral trabecular bone, were subsequently further divided into their medial and lateral halves through the posterolateral root of the posterior cruciate ligament, generating two independent tissue specimens for each patient tissue representing the lateral and medial halves of the same joint from a single patient to accommodate placement on standard 25 × 75 mm microscopy slides. This allowed for the comparison of medial OA sections (P1-P4, medial) to two distinct sets of ‘control’ tissues composed of i) site-matched non-arthritic cadaveric controls (C1-C4, medial) and ii) tissue-matched OA lateral tibial plateau halves (from the same OA patients, P1-P4, lateral). Lateral cadaveric control tissues were collected, processed, and imaged but resemble the cadaveric medial tissues in most cases.

### Tissue preparation and sectioning

Human tibial plateaus were fixed in 10% neutral buffered formalin (NBF) and incubated in an Ion Exchange Decalcification Unit (American Master Technologies) for 5–6 days until fully demineralized. Serial ethanol dehydration and paraffin infiltration and embedding were performed to generate FFPE tissue blocks. Tibial plateaus were sectioned onto standard pathology microscopy slides at 7 μm thickness in the coronal plane. Histological Safranin-O/Fast Green stain (Fig. S2) was used to identify regions of cartilage and cartilage damage in tissue sections. Unstained neighboring tissue sections were selected for Matrix-Assisted Laser Desorption Ionization – Mass Spectrometry Imaging (MALDI-MSI) preparation and imaging.

### Sample preparation for MALDI-MSI for ECM imaging

Formalin-fixed paraffin-embedded human tibial plateau tissue slides were prepared for enzymatic digestion using previously published protocols^46,48^ with important modifications in consideration of the large, ECM-rich tissue sections of the human tibial plateaus. This methodology was pioneered and published by Dr. Angel who demonstrated that the approach is highly reproducible across serial sections of FFPE tissues ( < 5% mean relative percent change across serial sections),^83^ efficiently extracts uniform peptide profiles from various tissue types,^84^ and have used the approach in many studies focused on cancer, where the biological variability is the largest factor in variation.^85^

The slides were heated at 60 °C for 1 h to melt off excess paraffin wax. After cooling, tissue sections were further deparaffinized by washing twice in xylene (3 min each). Tissue sections were washed and rehydrated by submerging in 100% ethanol twice (1 min each), once in a Carnoy’s solution (60% ethanol, 30% chloroform and 10% glacial acetic acid), to remove excess fat and wax, once in 95% ethanol (1 min), once in 70% ethanol (1 min), and twice in water (3 min each). After the rehydration series, slides were transferred to a slide mailer containing citraconic anhydride buffer (Thermo) at pH 3.0 for heat induced antigen/epitope retrieval. The slide mailer was sealed and gently arranged in a 60 °C oven so that submerged slides were positioned face up while the mailers were additionally submerged in a larger container (glass beaker) containing buffer that allowed for their orientation and provided an additional buffer reservoir, and left to incubate overnight, not exceeding 12 h. Slide mailers were then removed from the oven and allowed to cool on the bench top. Citraconic buffer was slowly exchanged with water, as to not impact the tissue, five times by removing half of the buffer via pipette and replacing it with water, prior to replacing it completely with water for the final solution exchange. Deglycosylation was achieved by applying a 0.1 μg/μL PNGase F solution (N-Zyme Scientifics) via the TM Sprayer M3 (HTX Imaging, Chapel Hill, NC, USA), followed by incubation in custom humid chambers for 2 h at 37 °C. After deglycosylation, slides were coated with a 7 mg/mL solution of alpha-cyano-4-hydroxycinnamic acid (CHCA) 50% acetonitrile, 0.1% trifluoracetic acid matrix solution via the TM Sprayer M3. After glycan imaging or deglycosylation, slides were washed carefully with 70% ethanol to remove the applied CHCA matrix, a high pH buffer (10 mmol/L Tris, pH 9, and low pH buffer, 10 mmol/L citraconic anhydride, pH 3, followed by distilled water to remove glycans and residual enzyme. Specific care was taken to ensure that serial enzymatic digestion post glycan imaging would not disrupt the integrity of the tissue slides.^46,47^ Following the wash, slides were vacuum dried in a desiccator and prepared for ECM peptide imaging performing a second epitope retrieval (10 mmol/L Tris, pH 9 overnight at 60 °C). ECM-targeted enzymatic digestion for proteomic imaging was completed utilizing collagenase III that featured an enzymatic preference to cleave at repeated amino acid Gly-Xxx-Pro (GXP) motifs within the triple helical region of collagens.^86,87^ While preferably targeting collagens, this enzyme formulation also allowed for the detection of several other ECM and ECM-related proteins (Table S2). The proteolytic enzyme pre-treatment was composed of a 0.1 μg/μL collagenase III (COLase3 (*Clostridium histolyticum*) Worthington Biochemical, Lakewood, NJ, USA) solution applied via the TM Sprayer M3, and incubated for 5 h in humid chambers at 37 °C. After incubation, slides were vacuum dried in a desiccator and coated with CHCA matrix (7 mg/mL solution of alpha-cyano-4-hydroxycinnamic acid (CHCA) 50% acetonitrile, 1.0% trifluoracetic acid). The CHCA matrix solution was spiked with [Glu1]-fibrinopeptide B human (GluFib) (Sigma-Aldrich, St Louis, MO, USA) for a total concentration of 200 fmol/L. After matrix drying for 30 min in a desiccator, the slides were rapidly immersed in cold 5 mmol/L ammonium phosphate, monobasic for <1 s and immediately dried in a desiccator.

### MS imaging data processing and statistical analysis

Image processing and mass spectrometry data analysis were completed in SCiLS Lab MVS v2024a Pro (Bruker Scientific, LLC, Bremen, Germany). During import, SCiLS lab background normalizes and resamples the raw data to ensure a common mass axis across the several thousand independent spectra acquisitions using a TIC preserving algorithm. This is accomplished through the SCiLS lab Import Wizard.^88^ Images generated by SCiLS for spatial representation of MS data are scaled such that the top 1% of most intense pixels are removed (hotspots) and the 100% value of the scale bar represents this cutoff intensity. After importation, each composite spectrum is further normalized to the glufibrinogen (GluFib) standard peptide at m/z 1 570.676 8 such that the remaining spectra have the same ion count in the peak interval (unit area under the peak) and the rest of each spectrum is scaled accordingly.^89^ Other normalization options available during the workflow, including normalization to the total ion current (TIC) or the root mean square (RMS) of signal intensity were explored but since this study involved the comparison of multiple samples that were imaged separately, to account for any possible differences between acquisitions, the external introduced GluFib normalization was chosen to standardize feature intensity across all imaged tissues. Backgrounded and normalized spectra are additionally filtered such that only the top 500 most abundant m/z features from each tissue are used in subsequent data analysis to avoid residual noise. This selected 500 peak threshold retained inherent biologic variability while removing the lower intensity peaks with high signal to noise ratios. These peaks were independently and automatically detected for each tissue and used to generate tissue regions through a Bisecting K-means method using the Segmentation operation within SCiLS Lab.^90^ This unbiased segmentation recognized regions of tissue that shared similar spectral profiles and grouped them together into regions. The number of hierarchical regions generated was set to the minimum required to identify cartilage regions from bone and to resolve subchondral trabecular bone against the background or marrow space areas. This unsupervised processing typically generated 4 to 6 tissue subregions often identifying multiple transitional bone subtypes. These generated regions are used to perform paired Discriminating Feature analysis^52,91,92^ within SCiLS Lab. These analyses processed all 30 000-40 000 captured spectra per imaged tissue (specific number of spectra differed by tissue size per sample) to identify candidate marker features (m/z) defining the difference between identified non-arthritic and diseased subchondral bone regions in both site-matched (cadaveric “healthy donor medial to OA medial tissues) and tissue-matched (OA patient lateral vs OA patient medial) comparisons. Candidate markers were considered if the area under the receiver operating curve (ROC) generated by the Discriminating Feature analysis for a given m/z was ≥0.85. Candidates were first generated by comparing one representative sample from the control group to the OA group. Candidates were later validated across the whole cohort by exporting intensity values for each candidate m/z out of SCiLS lab. Technical and computational limitations on file size prevented simultaneous analysis of numerous large files at once in SCiLS lab. To ensure then that candidate lists were not overly sample specific, similar sets of candidate peptides were generated using tissue sections from different individuals (Fig. S7). In addition to the automated tissue segments from SCiLS Lab, histologic stains of serial tissue sections were used to draw regions of interest (ROIs) manually. These ROIs included the diseased regions of subchondral bone to the edge of exposed cartilage on the medial side in OA patients or an equal distance of subchondral bone of the lateral side of the tissue matched joint (Fig. 5a), or ~2/5 the length of subchondral bone in cadaveric controls from the medial edge towards the center (the approximate distance of lost cartilage in medial OA samples) (Fig. S5). The individual pixel intensities by ROI for each candidate m/z feature were exported from SCiLS and the mean pixel intensity was calculated for each candidate feature per each ROI (patient or control sample). Peptide identifications were assigned to m/z features by searching the measured MS1 singly charged precursor ions (M + H) and their m/z value for each feature against previously generated peptide libraries assembled from other tissue types, including breast, cardiac, and lung tissues.^20,43,48^ Identifications were considered valid if the mass difference between the MALDI feature m/z and the peptide library were ≤2 mDa ( ~ 1.5 × 10^-5^) from the detected m/z value on the MS1 level in the MALDI-MSI to matching values in the separate databases that had been confirmed in greater detail in LC-MS/MS.

In some cases, detected m/z features had multiple potential peptide or protein matches in databases and so the multiple ID sequences are reported (Table S2), but overall statistical analysis considers features with multiple matches as a single candidate feature for reporting purposes. Further MS/MS is needed to confirm tissue-specific peptide sequence matches. In Results, only features with unique identifications are presented and discussed. MetaboAnalyst v5.0^93^ was used to generate three dimensional partial least squares-discriminant analysis (PLS-DA) plots using mean feature pixel intensity from the combined set of unique candidate features obtained through ROC analysis for each group comparison type. Although some candidate features were originally found from ROC analysis in one comparison or another (cadaveric Medial vs OA Medial or OA Medial vs OA Lateral), they exist as detectable features in all samples and statistical testing for group-wise comparisons between OA medial and the two control groups utilized the full feature list. Candidate m/z features were “validated” in group-wise comparisons using the sample mean intensity for individual candidate features from each independent tissue sample with Prism Version 10.1.1 (GraphPad) utilizing a paired or unpaired one-sided student’s *t*-test as appropriate (e.g., within patient tissue or between different individuals) with significance set a priori at *P* < 0.05. A one-sided *t*-test was selected given that intensity distributions from each ROI were right-skewed and intensity values cannot be negative values. Biological replicates of *n* = 4 for all groups.

### Mass spectrometry imaging

Mass spectrometric imaging data after proteolytic digestion with collagenase III was acquired using a Trapped Ion-Mobility Time-of-Flight Mass Spectrometer, the timsTOF fleX (Bruker Scientific, LLC, Bremen, Germany) equipped with a dual ESI/MALDI source. Samples were scanned across a MS1 mass range at m/z 600–4 000. The SmartBeam 3D 10 kHz laser was set to 20% power, scan range of 20 μm for X and Y and resulting field size of 24 μm for X and Y. 300 spectra were collected per pixel spot. Additional instrument parameters for the studies included: an ion transfer time of 75.0 μs, pre pulse storage time of 20 μs, a collision RF of 2 500 Vpp, a collision energy of 25 eV, an ion energy in the quadrupole of 5.0 eV, a TIMS funnel 1 RF of 400 Vpp, a TIMS funnel 2 RF of 500 Vpp and a multipole RF of 400 Vpp. After MS acquisition, the data was imported into SCiLS Lab MVS v2024a Pro (Bruker Scientific, LLC, Bremen, Germany). MSI generates spatial maps of the imaged tissue in which the intensities correspond to the abundance of a molecular feature (e.g., peptide) at the imaged location with a spot size of 20 μmol/L, with variable step-size resolution from adjacent pixels at 20 μmol/L laser step size to capture small regions in detail to a larger step sizes of 120 μmol/L or more to create images of the entire tissue section.

### Tissue preparation and electrospray ionization MS/MS

#### Spectral library building

After imaging, the MALDI matrix was removed by 70% ethanol wash. Tissue was removed from slides and fully digested in solution with the same collagenase III used to prepare slides for imaging. Excess enzyme removal and desalting of digested peptides were completed by sequential stage tip and ZipTip (Merk Millipore, Burlington MA) purification. Samples (100 ng) were loaded onto EvoTips Pure (Evosep, Odense, Denmark) following the manufacturer’s protocol. LC-MS/MS analyses were performed on an Evosep One liquid chromatography system (Evosep) coupled to a timsTOF HT mass spectrometer (Bruker, Bremen, Germany). The solvent system consisted of 0.1% formic acid (FA) in water (solvent A) and 0.1% FA in ACN (solvent B). Peptides were eluted on a PepSep C_18_ analytical column (150 µm x 15 cm, 1.5 µm particle size; Bruker) using the 30 SPD method (44-min gradient length, 500 nL/min flow rate). A zero-dead volume emitter was installed in the nano-electrospray source (CaptiveSpray source, Bruker Daltonics) and the source parameters were set as follows: capillary voltage 1 600 V, dry gas 3 L/min, and dry temperature 180 °C. Each sample was acquired in data-dependent acquisition (DDA) - parallel accumulation–serial fragmentation (PASEF)^94^ mode with 4 PASEF MS/MS ramps and with the trapped-ion mobility (TIMS) mobilogram inclusion parallelogram expanded to include monocharged precursor ions (Table S3). For DDA-PASEF with TIMS on, MS1 and MS2 spectra were acquired over an m/z range of 100–1 700 m/z and an ion mobility range of 0.85–1.70 Vs/cm^2^. Precursor ions were selected fragmentation with charge state from 0 to 5, target intensity of 12 500, and intensity threshold of 500. The dual TIMS analyzer was operated in a 100% duty cycle with equal accumulation time and ramp time of 150 ms each, for a total cycle time of 0.78 s. The collision energy was defined as a linear function of mobility starting from 20 eV at 1/K0 = 0.6 Vs/cm^2^ to 59 eV at 1/K0 = 1.6 Vs/cm^2^. For calibration of ion mobility dimension, three ions of Agilent ESI-Low Tuning Mix ions were selected (m/z [Th], 1/K0 [Vs/cm^2^]: 622.028 9, 0.991 5; 922.009 7, 1.198 6; 1 221.990 6, 1.139 34). Spectral libraries from acquisitions were generated utilizing SpectroMine version 4.0 (Biognosys AG, Schlieren, Switzerland) using a nonspecific enzymatic digestion with peptide length of 7-52 amino acids, with fixed cysteine carbamidomethyl modifications and variable N-terminal protein acetylation and oxidation of methionine and proline. Protein, peptide, and peptide-spectrum match (PSM) FDR scores were set to 0.01. The PSM as reported by SpectroMine is a scoring function that assigns a numerical value to a peptide-spectrum pair (*P,S*) expressing the likelihood that the fragmentation of a peptide with sequence *P* is recorded in the experimental mass spectrum *S*.^95^

Searches were performed against a Human UniProtKB/SwissProt reviewed database downloaded from UniProt on April 3, 2023, containing 1 032 entries of proteins with “Extracellular Matrix” GO cellular component annotation. Finally, the best 3-6 fragments per peptide were kept. MS/MS spectra for hydroxyproline containing peptides were manually inspected and verified in Skyline-daily v21.2.1.535 (University of Washington, WA, USA).^96^ The final library contained 543 peptides, 320 of which contained hydroxyproline modifications, all corresponding to 58 protein groups, all spectra were manually reviewed. For peptides where DDA analysis was unable to specifically determine HYP modification site location due to multiple potential prolines within a peptide, Table S2 lists the PTM site score probabilities as determined by MaxQuant for each modified peptide as reported by other databases.^97^

#### Synovial fluid analysis

Synovial fluid samples were collected by and purchased from StemBioSys (San Antonio, TX). 1 mL of synovial fluid aliquots from each donor were prepared for LC-MS/MS Data Independent Acquisition (DIA). First, samples were subjected to protein precipitation using the ProteoExtract Protein Precipitation Kit (Sigma-Aldrich, St. Louis, MO) to remove hyaluronic acid. After precipitation, samples were resuspended in 0.2% FA in H_2_O and prepared for tryptic digestion using S-TRAP column (ProtFi, Fairport, NY) protocols with trypsin. 1 μg of each digested sample was acquired in DIA mode (ZenoSWATH DIA)^98,99^ as double technical replicates using a 120 min microflow gradient with a Waters M-Class HPLC (Waters, Massachusetts, USA) coupled online to a ZenoTOF 7600 system (SCIEX, Redwood City, CA) with an OptiFlow Turbo V Ion Source using a microelectrode(1–10 μL/min) with the Zenotrap enabled. The survey MS1 spectra were acquired from 395–1 005 m/z with a 100-ms accumulation time. The MS1 accumulation time was set to 25 ms and 80 variable windows were used to collect MS/MS spectra, resulting in a total cycle time of 2.5 s. All data files were processed in Spectronaut (version16.0.220524.5300; Biognosys) using DirectDIA using the *homo sapiens* reference proteome with 92 931 entries (UniProtKB-TrEMBL), accessed on 12/13/2023. Relative protein abundance changes were compared in a statistically relevant manner using the Storey method with paired *t*-tests and *P*-values corrected for multiple testing by applying group wise testing corrections.^100^ A statistical cut-off of |log_2_(FC) | ≥ 0.58 and *q*-value ≤ 0.01 was applied to identify significant changes in protein abundance.

## Supplementary information

Supplementary Figure 1

Supplementary Figure 2

Supplementary Figure 3

Supplementary Figure 4

Supplementary Figure 5

Supplementary Figure 6

Supplementary Figure 7

Supplementary Figure 8

Supplementary Figure 9

Supplementary Figure 10

Supplementary Figure 11

Supplementary Figure 12

Supplementary Figure 13

Supplementary Figure 14

Supplementary Figure 14

Supplementary Table 1

Supplementary Table 2

Supplementary Table 3

Supplementary Table 4

Supplementary Table 5

## Data Availability

The human spatial proteomic datasets have been uploaded to the Center for Computational Mass Spectrometry MassIVE repository at UCSD and can be accessed using the following link: https://massive.ucsd.edu/ProteoSAFe/private-dataset.jsp?task=c8ee2b4247994c4396007e1cffd0bcfb MassIVE ID number: **MSV000094448; Proteome Xchange number: PXD051135**.
