# Supplementary material for: Tissue and extracellular matrix remodeling of the subchondral bone during osteoarthritis of knee joints as revealed by spatial mass spectrometry imaging
Source: Bone Res. 2026 Jan 26;14:14. doi: 10.1038/s41413-025-00495-0 (PMC12835079; doi:10.1038/s41413-025-00495-0)
Supplement: Supplementary file 1 — Supplementary Figure 1 [file 41413_2025_495_MOESM1_ESM.pptx]

## Slide 1
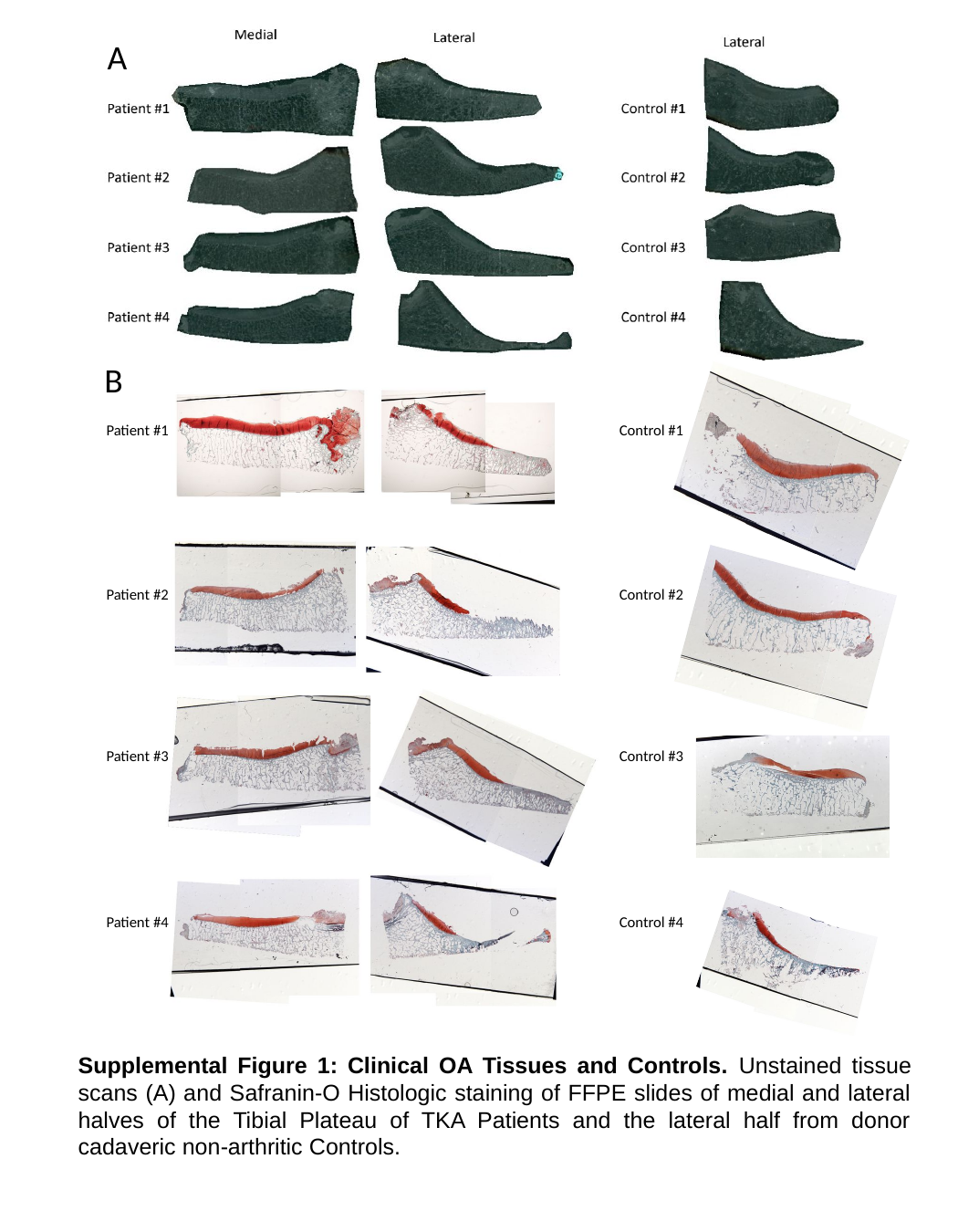

A
B
Patient #1
Control #1
Patient #2
Control #2
Patient #3
Control #3
Patient #4
Control #4
Supplemental Figure 1: Clinical OA Tissues and Controls. Unstained tissue scans (A) and Safranin-O Histologic staining of FFPE slides of medial and lateral halves of the Tibial Plateau of TKA Patients and the lateral half from donor cadaveric non-arthritic Controls.
