# Supplementary material for: Tissue and extracellular matrix remodeling of the subchondral bone during osteoarthritis of knee joints as revealed by spatial mass spectrometry imaging
Source: Bone Res. 2026 Jan 26;14:14. doi: 10.1038/s41413-025-00495-0 (PMC12835079; doi:10.1038/s41413-025-00495-0)
Supplement: Supplementary file 2 — Supplementary Figure 2 [file 41413_2025_495_MOESM2_ESM.pptx]

## Slide 1
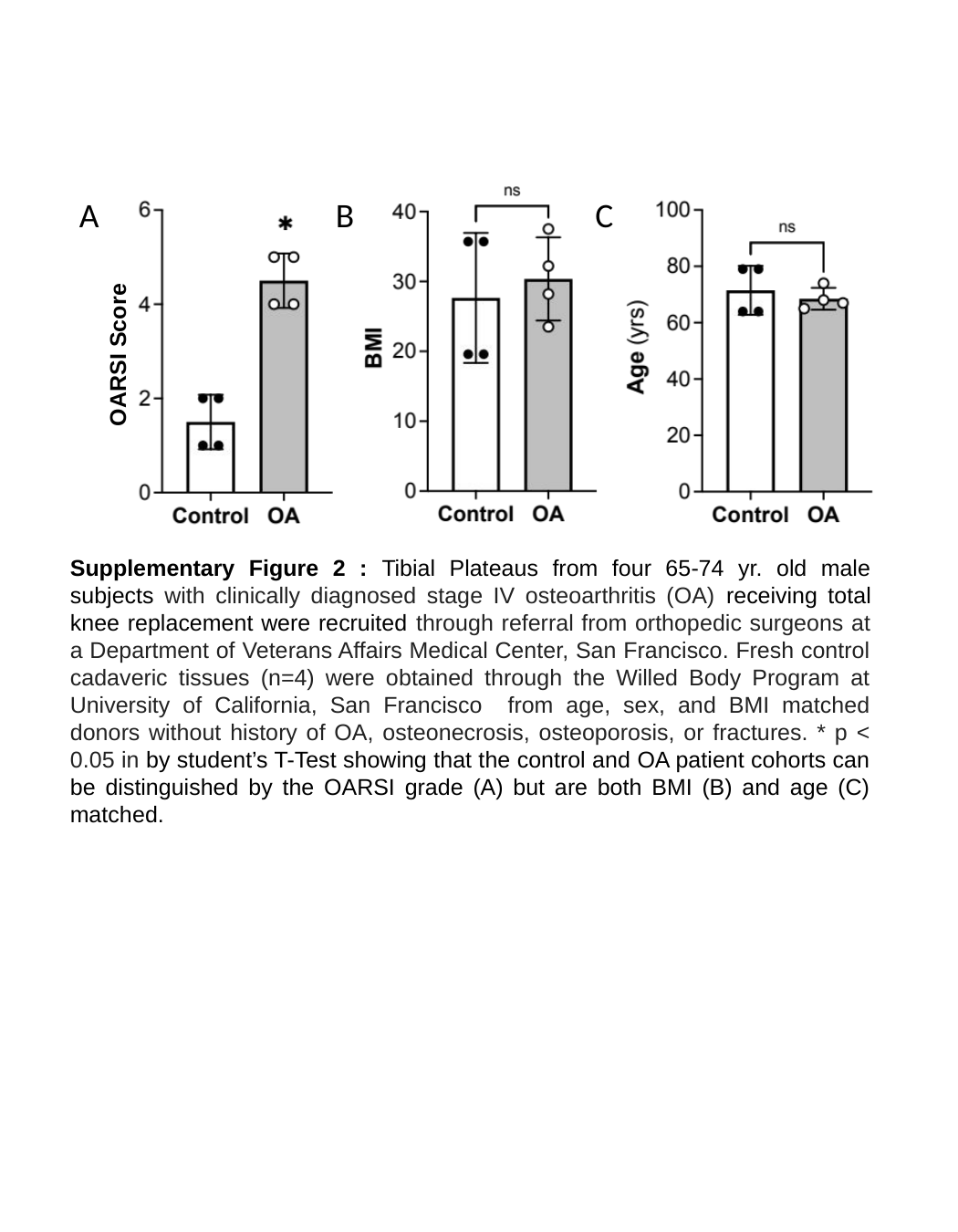

A
B
C
OARSI Score
Supplementary Figure 2 : Tibial Plateaus from four 65-74 yr. old male subjects with clinically diagnosed stage IV osteoarthritis (OA) receiving total knee replacement were recruited through referral from orthopedic surgeons at a Department of Veterans Affairs Medical Center, San Francisco. Fresh control cadaveric tissues (n=4) were obtained through the Willed Body Program at University of California, San Francisco from age, sex, and BMI matched donors without history of OA, osteonecrosis, osteoporosis, or fractures. * p < 0.05 in by student’s T-Test showing that the control and OA patient cohorts can be distinguished by the OARSI grade (A) but are both BMI (B) and age (C) matched.
