# Supplementary material for: Tissue and extracellular matrix remodeling of the subchondral bone during osteoarthritis of knee joints as revealed by spatial mass spectrometry imaging
Source: Bone Res. 2026 Jan 26;14:14. doi: 10.1038/s41413-025-00495-0 (PMC12835079; doi:10.1038/s41413-025-00495-0)
Supplement: Supplementary file 3 — Supplementary Figure 3 [file 41413_2025_495_MOESM3_ESM.pptx]

## Slide 1
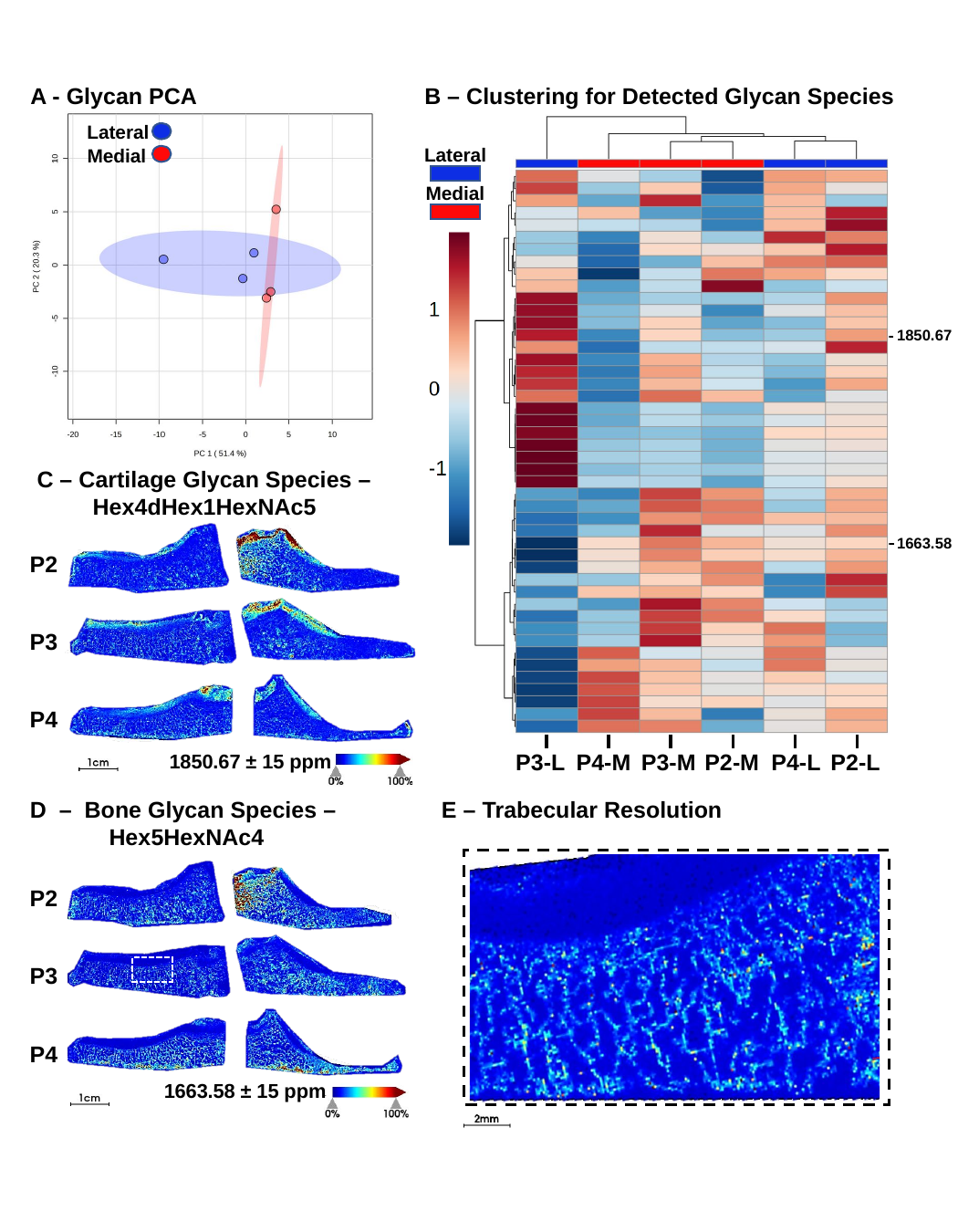

A - Glycan PCA
B – Clustering for Detected Glycan Species
Lateral
Medial
Lateral
Medial
1850.67
C – Cartilage Glycan Species –
Hex4dHex1HexNAc5
1850.67 ± 15 ppm
1663.58
P2
P3
P4
P3-L
P4-M
P3-M
P2-M
P4-L
P2-L
E – Trabecular Resolution
D – Bone Glycan Species –
 Hex5HexNAc4
1663.58 ± 15 ppm
P2
P3
P4

## Slide 2
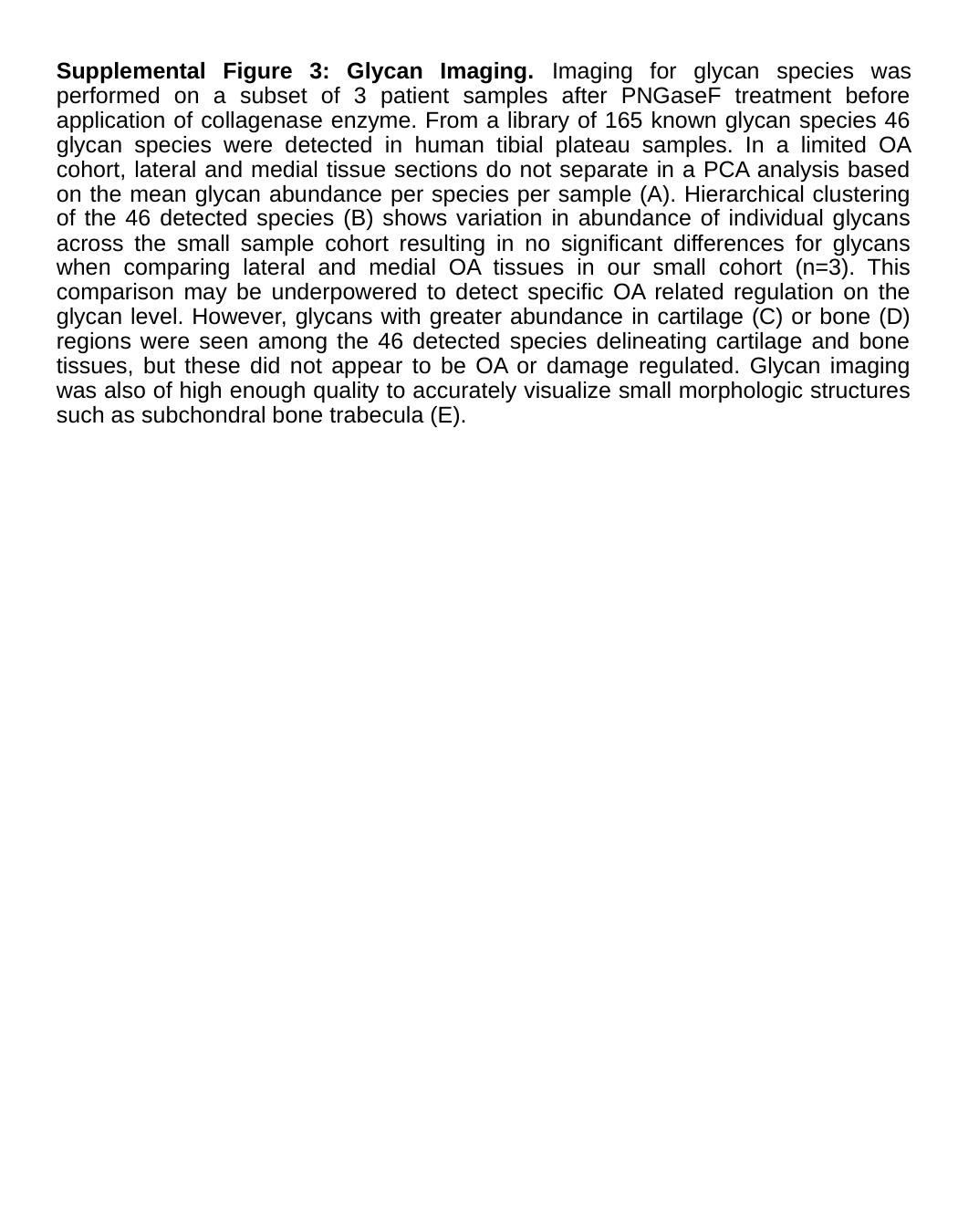

Supplemental Figure 3: Glycan Imaging. Imaging for glycan species was performed on a subset of 3 patient samples after PNGaseF treatment before application of collagenase enzyme. From a library of 165 known glycan species 46 glycan species were detected in human tibial plateau samples. In a limited OA cohort, lateral and medial tissue sections do not separate in a PCA analysis based on the mean glycan abundance per species per sample (A). Hierarchical clustering of the 46 detected species (B) shows variation in abundance of individual glycans across the small sample cohort resulting in no significant differences for glycans when comparing lateral and medial OA tissues in our small cohort (n=3). This comparison may be underpowered to detect specific OA related regulation on the glycan level. However, glycans with greater abundance in cartilage (C) or bone (D) regions were seen among the 46 detected species delineating cartilage and bone tissues, but these did not appear to be OA or damage regulated. Glycan imaging was also of high enough quality to accurately visualize small morphologic structures such as subchondral bone trabecula (E).
