# Supplementary material for: Tissue and extracellular matrix remodeling of the subchondral bone during osteoarthritis of knee joints as revealed by spatial mass spectrometry imaging
Source: Bone Res. 2026 Jan 26;14:14. doi: 10.1038/s41413-025-00495-0 (PMC12835079; doi:10.1038/s41413-025-00495-0)
Supplement: Supplementary file 4 — Supplementary Figure 4 [file 41413_2025_495_MOESM4_ESM.pptx]

## Slide 1
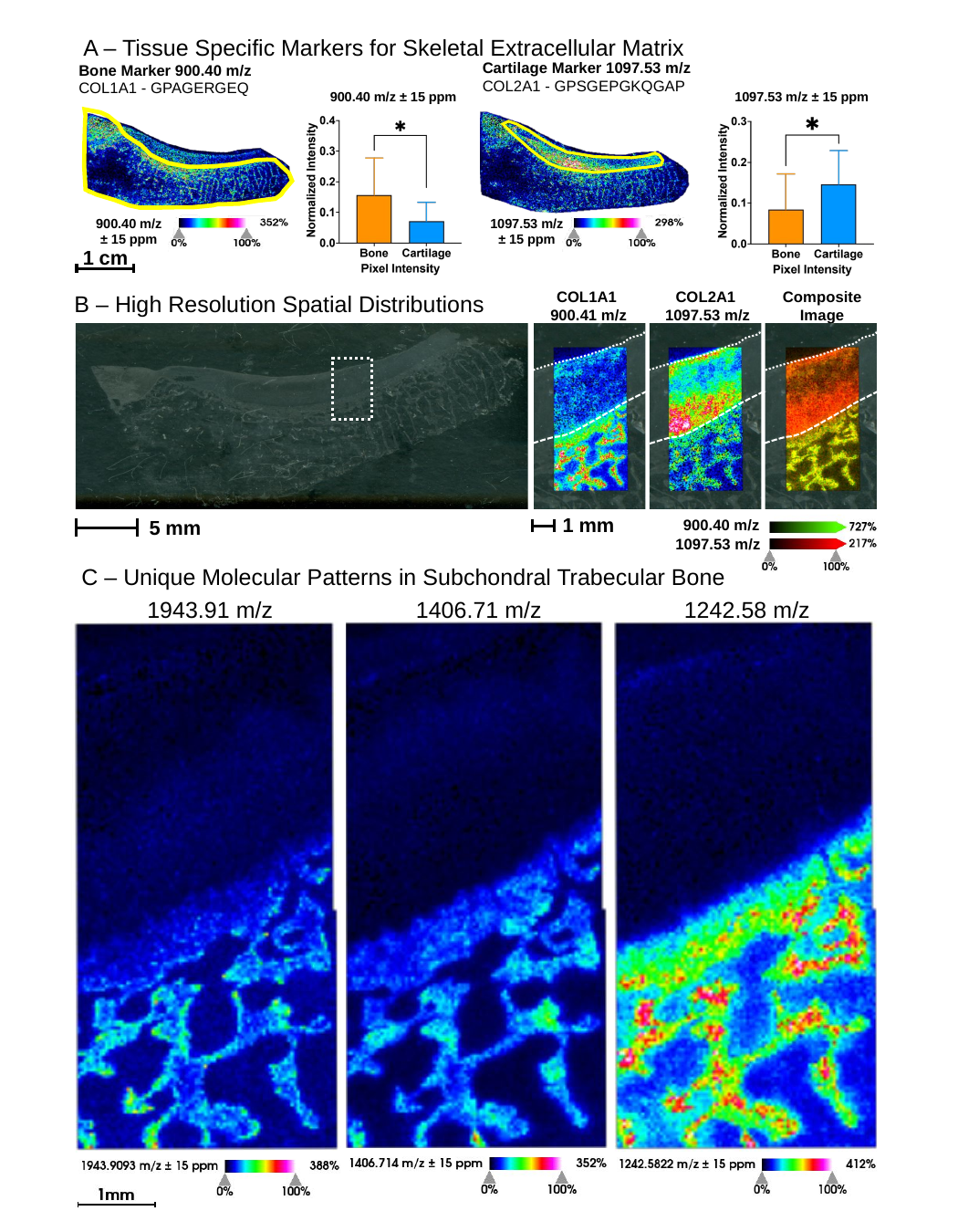

A – Tissue Specific Markers for Skeletal Extracellular Matrix
Cartilage Marker 1097.53 m/z
COL2A1 - GPSGEPGKQGAP
Bone Marker 900.40 m/z
COL1A1 - GPAGERGEQ
900.40 m/z ± 15 ppm
1097.53 m/z ± 15 ppm
900.40 m/z
± 15 ppm
1097.53 m/z
± 15 ppm
1 cm
COL1A1
900.41 m/z
COL2A1
1097.53 m/z
Composite
Image
B – High Resolution Spatial Distributions
1 mm
 900.40 m/z
5 mm
1097.53 m/z
C – Unique Molecular Patterns in Subchondral Trabecular Bone
1406.71 m/z
1242.58 m/z
1943.91 m/z

## Slide 2
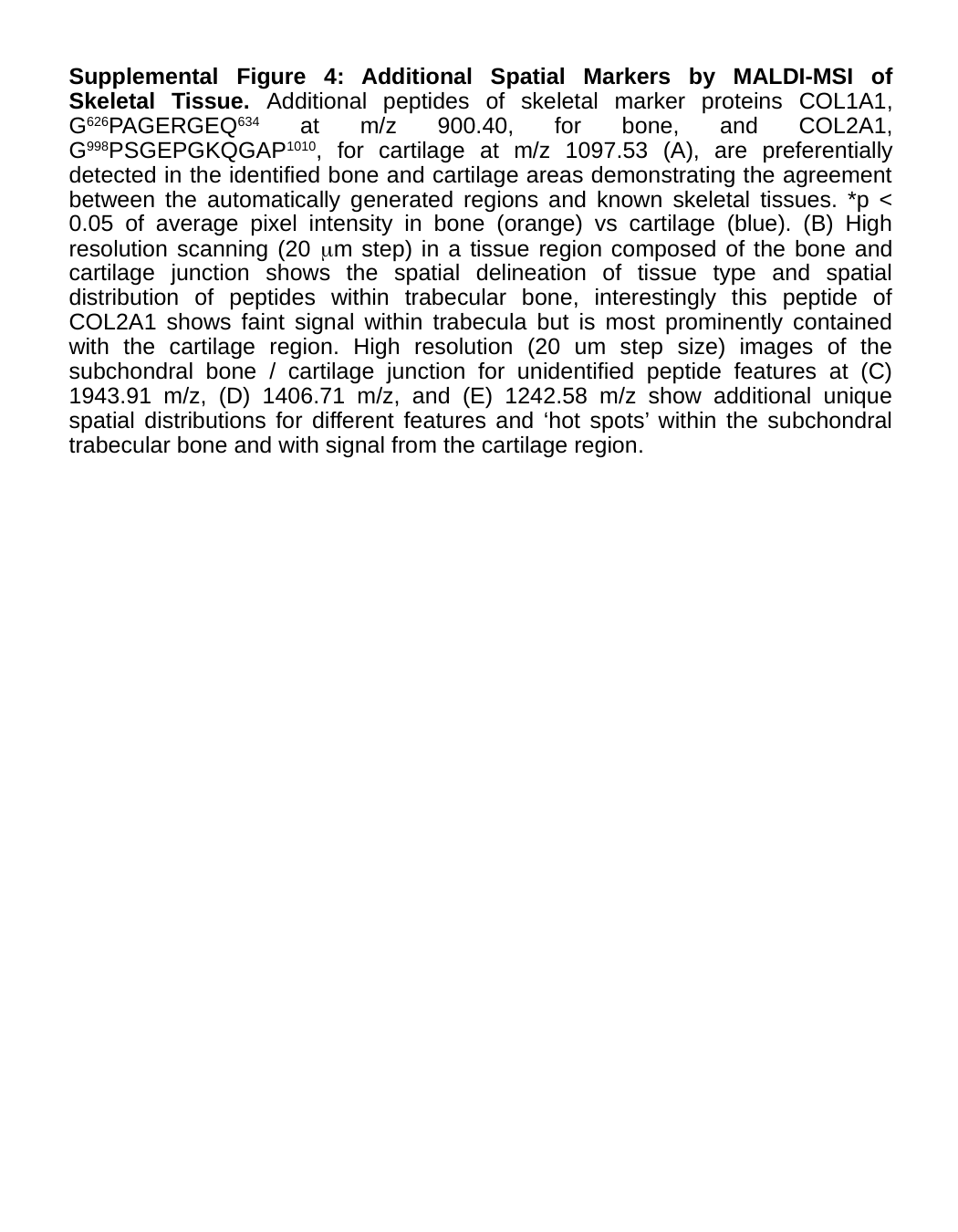

Supplemental Figure 4: Additional Spatial Markers by MALDI-MSI of Skeletal Tissue. Additional peptides of skeletal marker proteins COL1A1, G626PAGERGEQ634 at m/z 900.40, for bone, and COL2A1, G998PSGEPGKQGAP1010, for cartilage at m/z 1097.53 (A), are preferentially detected in the identified bone and cartilage areas demonstrating the agreement between the automatically generated regions and known skeletal tissues. *p < 0.05 of average pixel intensity in bone (orange) vs cartilage (blue). (B) High resolution scanning (20 μm step) in a tissue region composed of the bone and cartilage junction shows the spatial delineation of tissue type and spatial distribution of peptides within trabecular bone, interestingly this peptide of COL2A1 shows faint signal within trabecula but is most prominently contained with the cartilage region. High resolution (20 um step size) images of the subchondral bone / cartilage junction for unidentified peptide features at (C) 1943.91 m/z, (D) 1406.71 m/z, and (E) 1242.58 m/z show additional unique spatial distributions for different features and ‘hot spots’ within the subchondral trabecular bone and with signal from the cartilage region.
