# Supplementary material for: Tissue and extracellular matrix remodeling of the subchondral bone during osteoarthritis of knee joints as revealed by spatial mass spectrometry imaging
Source: Bone Res. 2026 Jan 26;14:14. doi: 10.1038/s41413-025-00495-0 (PMC12835079; doi:10.1038/s41413-025-00495-0)
Supplement: Supplementary file 5 — Supplementary Figure 5 [file 41413_2025_495_MOESM5_ESM.pptx]

## Slide 1
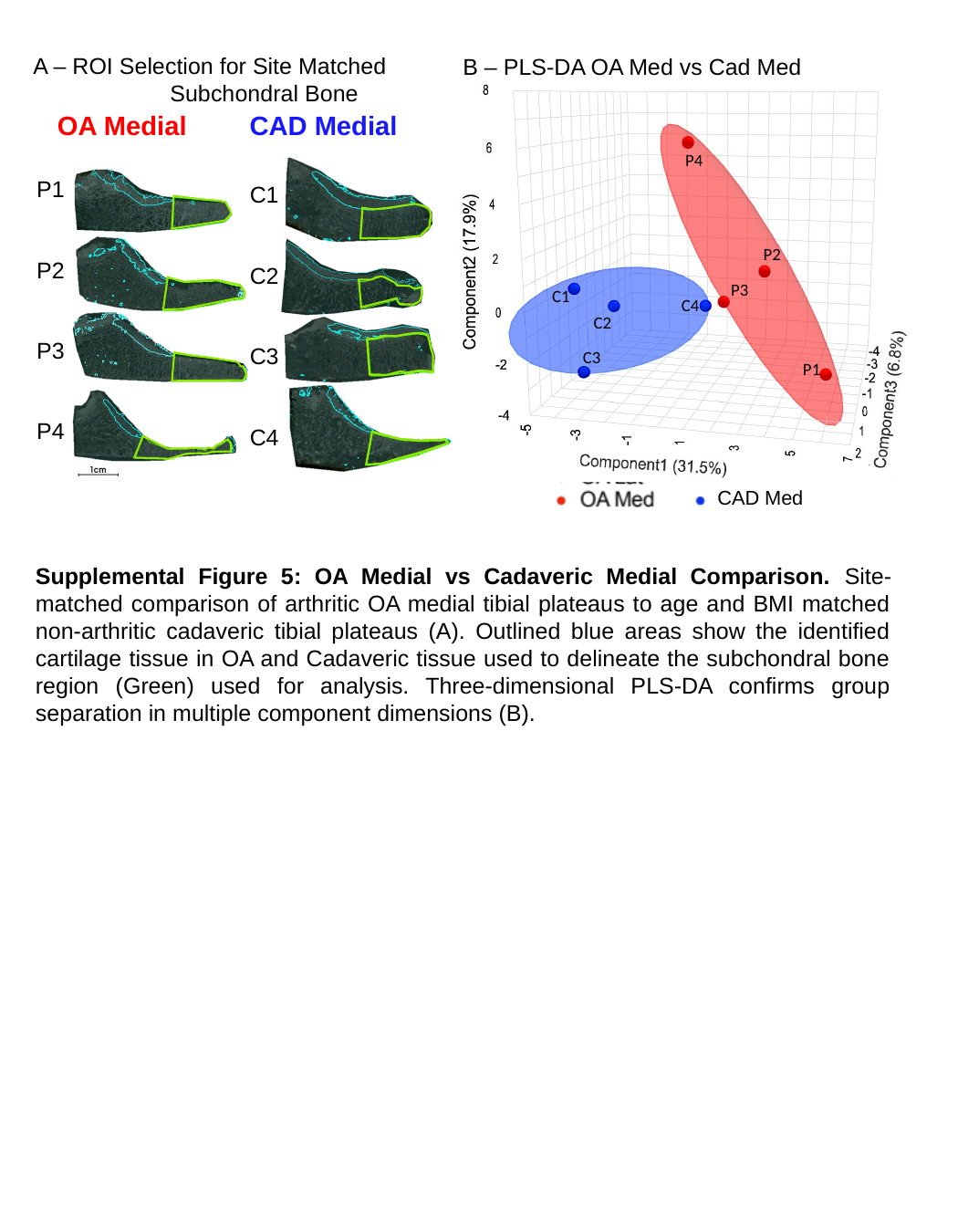

A – ROI Selection for Site Matched 	Subchondral Bone
B – PLS-DA OA Med vs Cad Med
P4
P2
P3
C1
C4
C2
C3
P1
CAD Medial
OA Medial
C1
C2
C3
C4
P1
P2
P3
P4
CAD Med
Supplemental Figure 5: OA Medial vs Cadaveric Medial Comparison. Site-matched comparison of arthritic OA medial tibial plateaus to age and BMI matched non-arthritic cadaveric tibial plateaus (A). Outlined blue areas show the identified cartilage tissue in OA and Cadaveric tissue used to delineate the subchondral bone region (Green) used for analysis. Three-dimensional PLS-DA confirms group separation in multiple component dimensions (B).
