# Supplementary material for: Tissue and extracellular matrix remodeling of the subchondral bone during osteoarthritis of knee joints as revealed by spatial mass spectrometry imaging
Source: Bone Res. 2026 Jan 26;14:14. doi: 10.1038/s41413-025-00495-0 (PMC12835079; doi:10.1038/s41413-025-00495-0)
Supplement: Supplementary file 6 — Supplementary Figure 6 [file 41413_2025_495_MOESM6_ESM.pptx]

## Slide 1
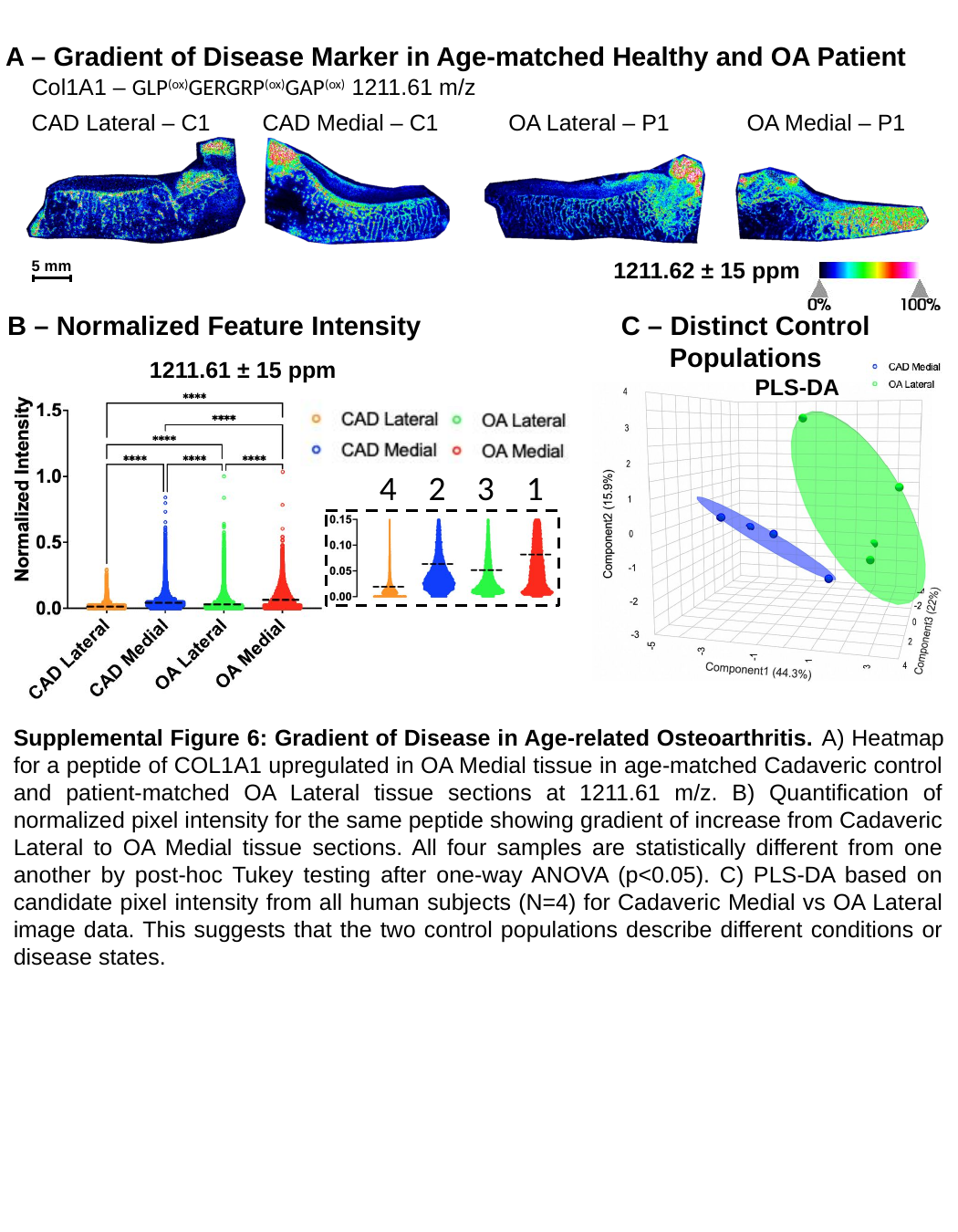

A – Gradient of Disease Marker in Age-matched Healthy and OA Patient
Col1A1 – GLP(ox)GERGRP(ox)GAP(ox) 1211.61 m/z
CAD Lateral – C1
CAD Medial – C1
OA Lateral – P1
OA Medial – P1
1211.62 ± 15 ppm
5 mm
B – Normalized Feature Intensity
C – Distinct Control Populations
1211.61 ± 15 ppm
PLS-DA
4
2
3
1
Supplemental Figure 6: Gradient of Disease in Age-related Osteoarthritis. A) Heatmap for a peptide of COL1A1 upregulated in OA Medial tissue in age-matched Cadaveric control and patient-matched OA Lateral tissue sections at 1211.61 m/z. B) Quantification of normalized pixel intensity for the same peptide showing gradient of increase from Cadaveric Lateral to OA Medial tissue sections. All four samples are statistically different from one another by post-hoc Tukey testing after one-way ANOVA (p<0.05). C) PLS-DA based on candidate pixel intensity from all human subjects (N=4) for Cadaveric Medial vs OA Lateral image data. This suggests that the two control populations describe different conditions or disease states.
