# Supplementary material for: Tissue and extracellular matrix remodeling of the subchondral bone during osteoarthritis of knee joints as revealed by spatial mass spectrometry imaging
Source: Bone Res. 2026 Jan 26;14:14. doi: 10.1038/s41413-025-00495-0 (PMC12835079; doi:10.1038/s41413-025-00495-0)
Supplement: Supplementary file 7 — Supplementary Figure 7 [file 41413_2025_495_MOESM7_ESM.pptx]

## Slide 1
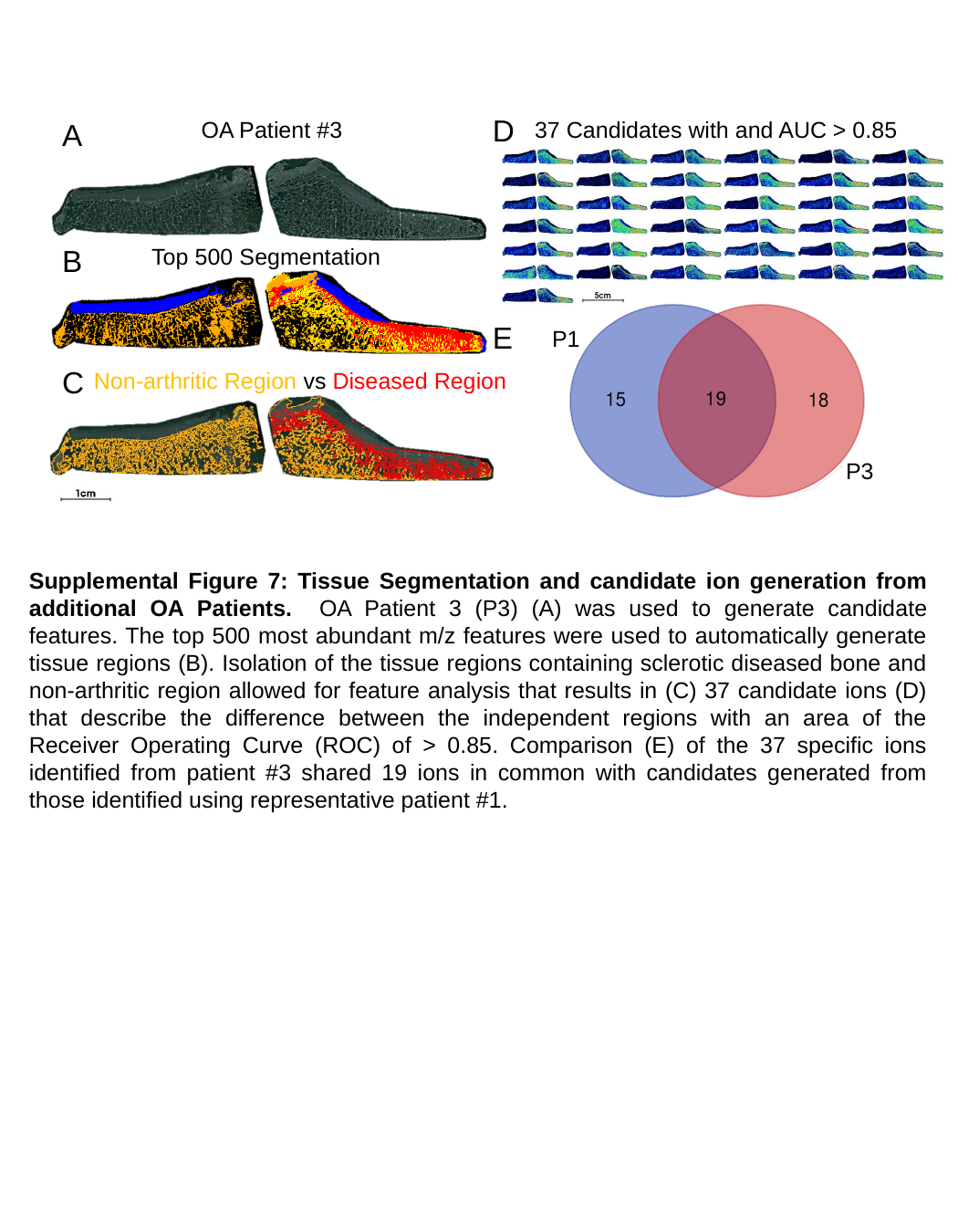

D
A
OA Patient #3
37 Candidates with and AUC > 0.85
B
Top 500 Segmentation
E
P1
C
Non-arthritic Region vs Diseased Region
P3
Supplemental Figure 7: Tissue Segmentation and candidate ion generation from additional OA Patients. OA Patient 3 (P3) (A) was used to generate candidate features. The top 500 most abundant m/z features were used to automatically generate tissue regions (B). Isolation of the tissue regions containing sclerotic diseased bone and non-arthritic region allowed for feature analysis that results in (C) 37 candidate ions (D) that describe the difference between the independent regions with an area of the Receiver Operating Curve (ROC) of > 0.85. Comparison (E) of the 37 specific ions identified from patient #3 shared 19 ions in common with candidates generated from those identified using representative patient #1.
