# Supplementary material for: Tissue and extracellular matrix remodeling of the subchondral bone during osteoarthritis of knee joints as revealed by spatial mass spectrometry imaging
Source: Bone Res. 2026 Jan 26;14:14. doi: 10.1038/s41413-025-00495-0 (PMC12835079; doi:10.1038/s41413-025-00495-0)
Supplement: Supplementary file 8 — Supplementary Figure 8 [file 41413_2025_495_MOESM8_ESM.pptx]

## Slide 1
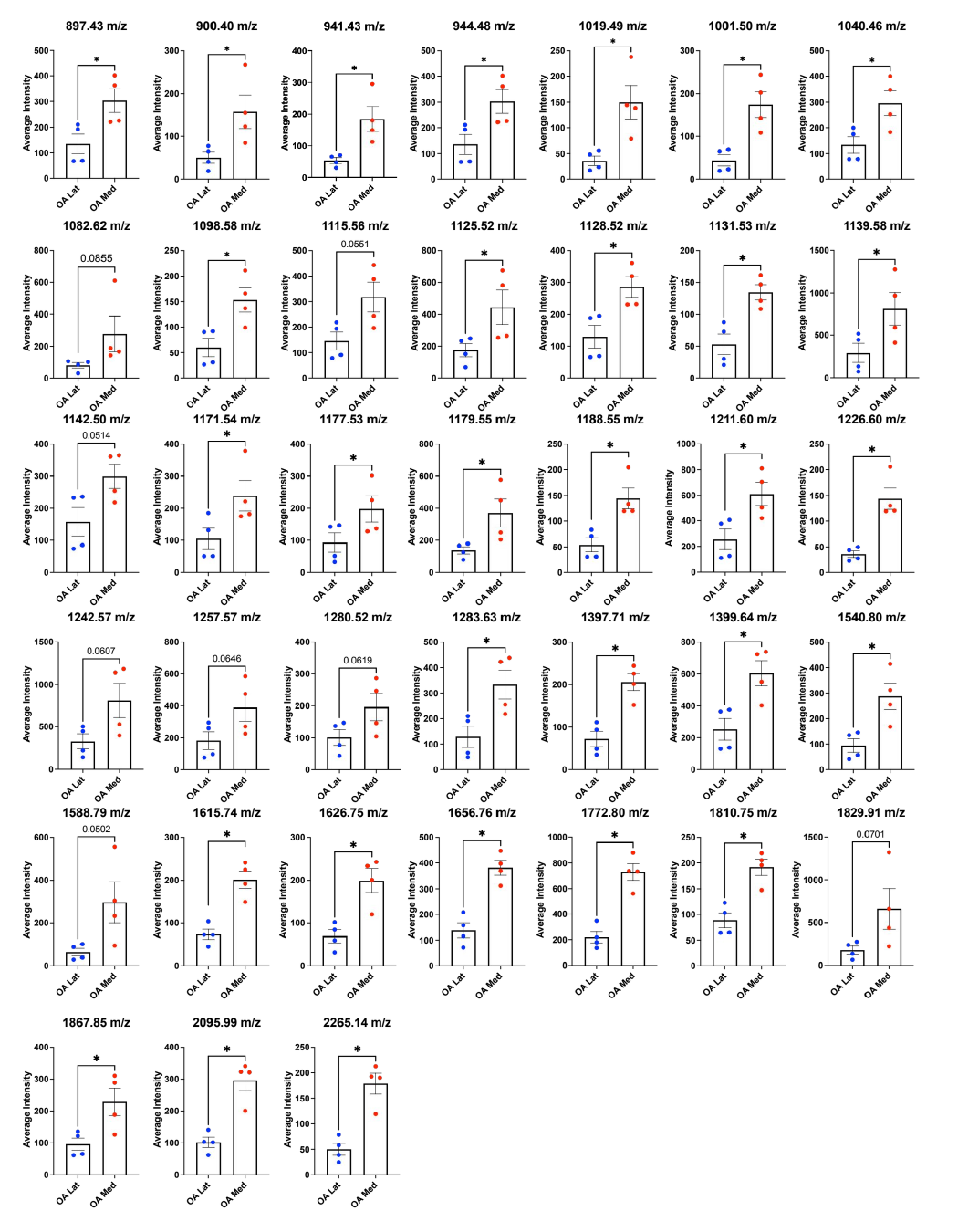

## Slide 2
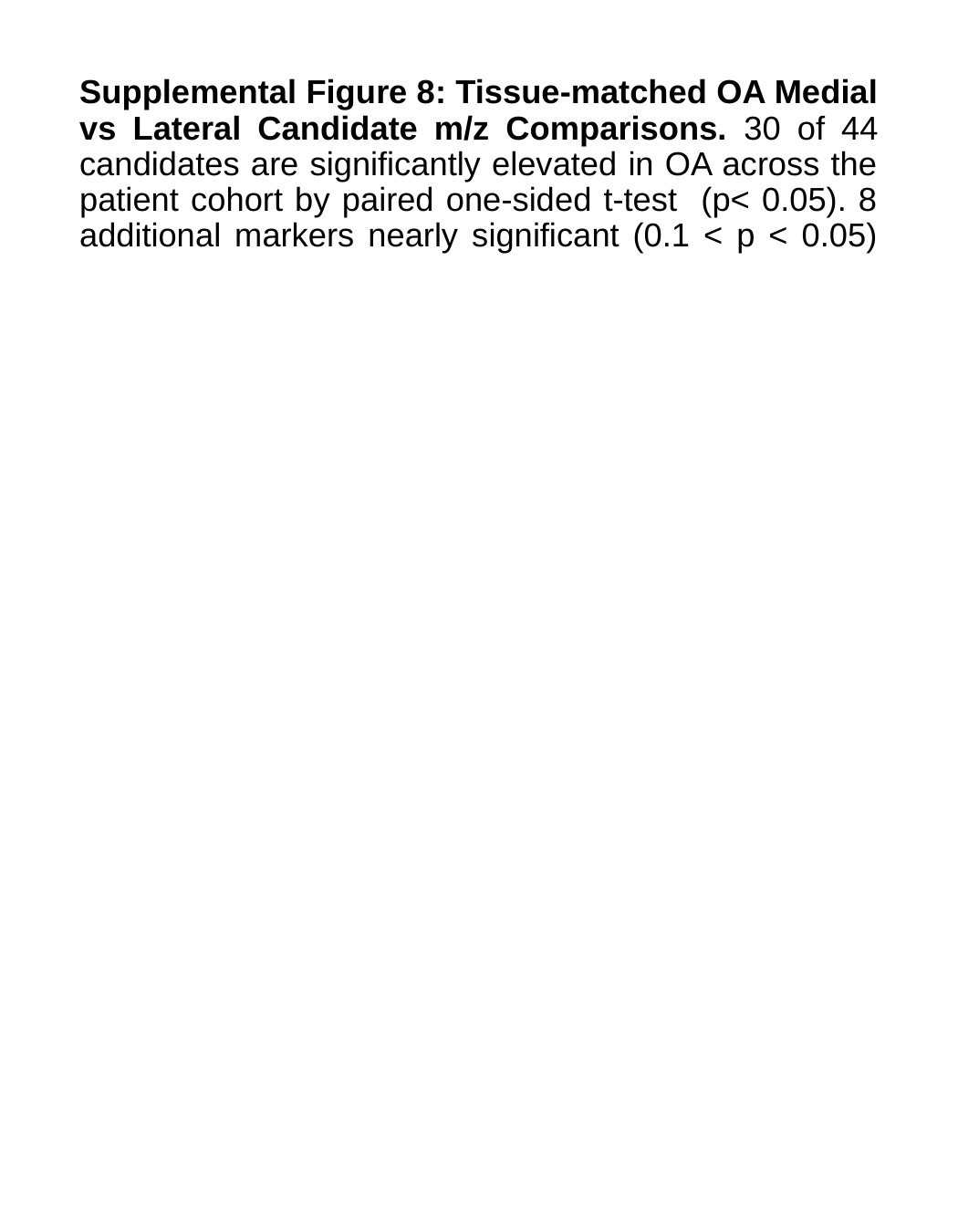

# Supplemental Figure 8: Tissue-matched OA Medial vs Lateral Candidate m/z Comparisons. 30 of 44 candidates are significantly elevated in OA across the patient cohort by paired one-sided t-test (p< 0.05). 8 additional markers nearly significant (0.1 < p < 0.05)
