# Supplementary material for: Tissue and extracellular matrix remodeling of the subchondral bone during osteoarthritis of knee joints as revealed by spatial mass spectrometry imaging
Source: Bone Res. 2026 Jan 26;14:14. doi: 10.1038/s41413-025-00495-0 (PMC12835079; doi:10.1038/s41413-025-00495-0)
Supplement: Supplementary file 9 — Supplementary Figure 9 [file 41413_2025_495_MOESM9_ESM.pptx]

## Slide 1
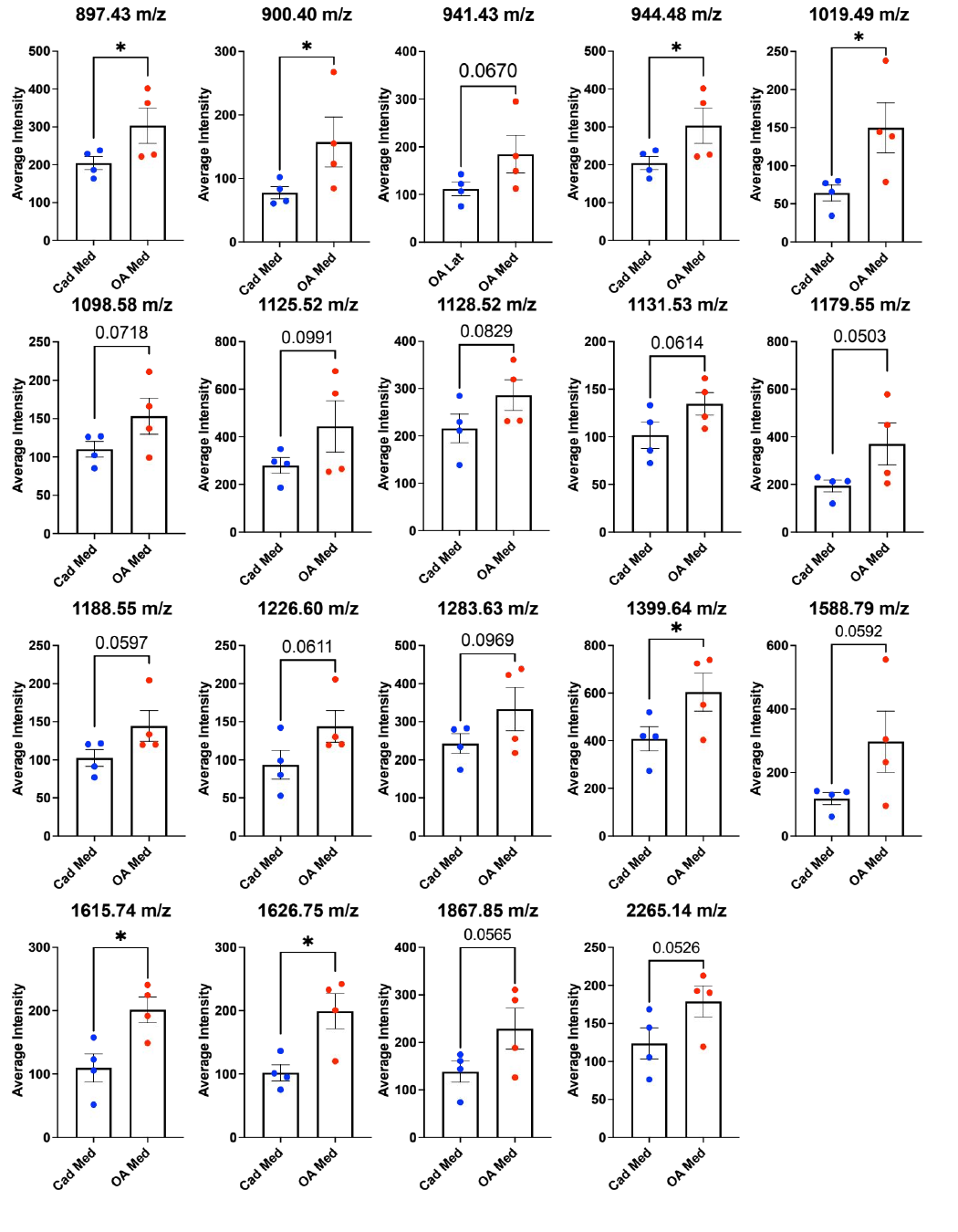

## Slide 2
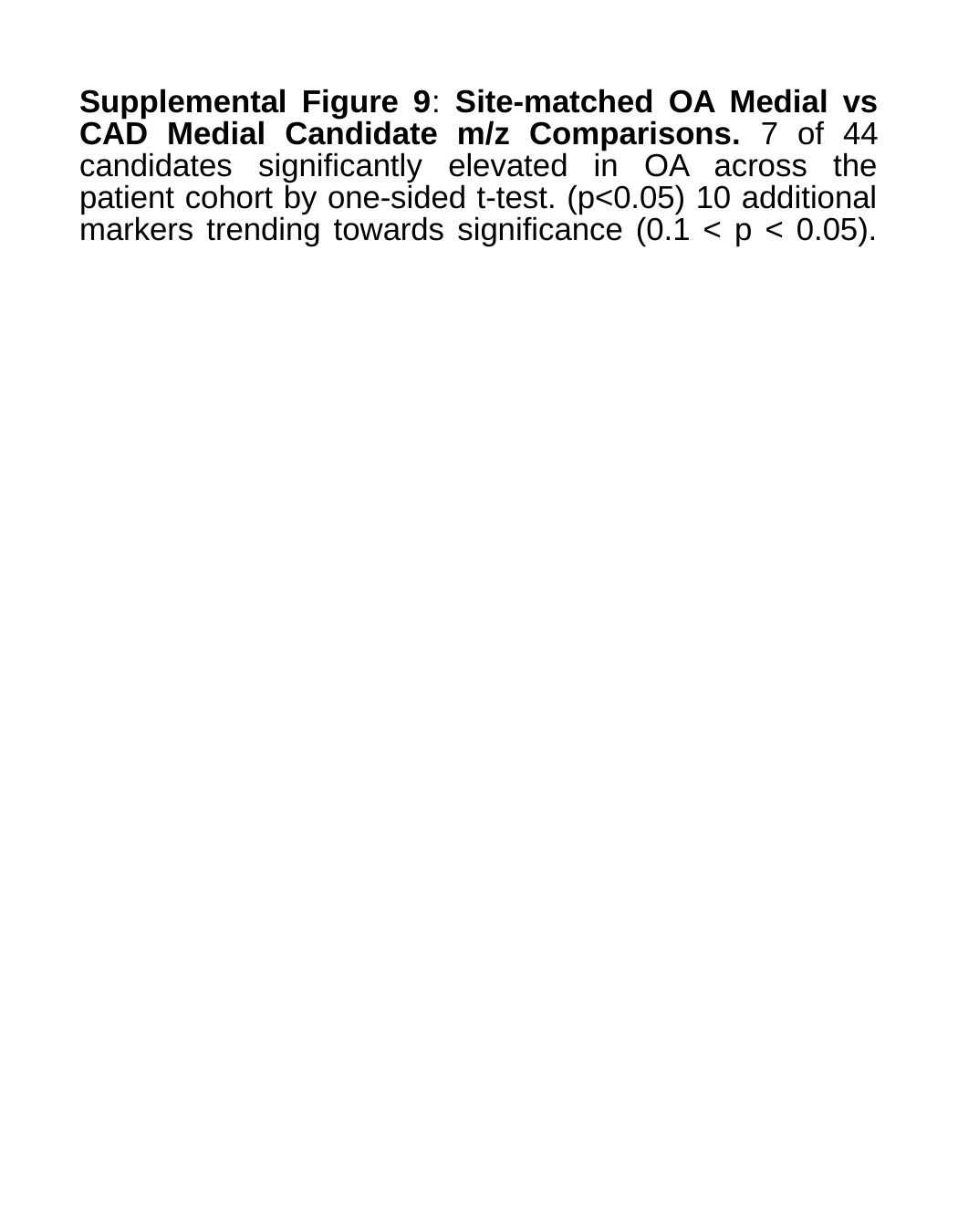

# Supplemental Figure 9: Site-matched OA Medial vs CAD Medial Candidate m/z Comparisons. 7 of 44 candidates significantly elevated in OA across the patient cohort by one-sided t-test. (p<0.05) 10 additional markers trending towards significance (0.1 < p < 0.05).
