# Supplementary material for: Tissue and extracellular matrix remodeling of the subchondral bone during osteoarthritis of knee joints as revealed by spatial mass spectrometry imaging
Source: Bone Res. 2026 Jan 26;14:14. doi: 10.1038/s41413-025-00495-0 (PMC12835079; doi:10.1038/s41413-025-00495-0)
Supplement: Supplementary file 10 — Supplementary Figure 10 [file 41413_2025_495_MOESM10_ESM.pptx]

## Slide 1
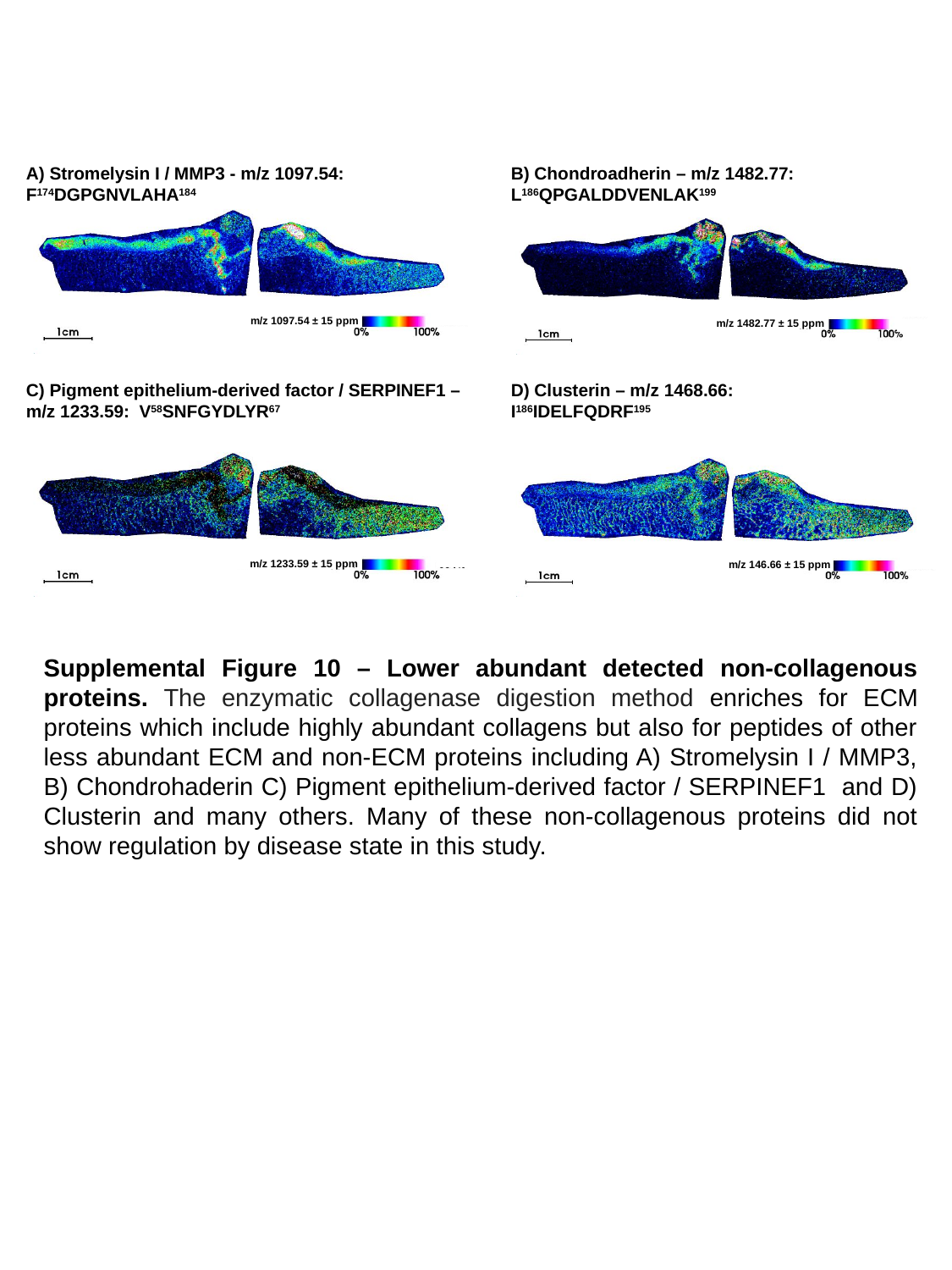

A) Stromelysin I / MMP3 - m/z 1097.54: F174DGPGNVLAHA184
B) Chondroadherin – m/z 1482.77: L186QPGALDDVENLAK199
m/z 1097.54 ± 15 ppm
m/z 1482.77 ± 15 ppm
C) Pigment epithelium-derived factor / SERPINEF1 – m/z 1233.59: V58SNFGYDLYR67
D) Clusterin – m/z 1468.66: I186IDELFQDRF195
m/z 1233.59 ± 15 ppm
m/z 146.66 ± 15 ppm
Supplemental Figure 10 – Lower abundant detected non-collagenous proteins. The enzymatic collagenase digestion method enriches for ECM proteins which include highly abundant collagens but also for peptides of other less abundant ECM and non-ECM proteins including A) Stromelysin I / MMP3, B) Chondrohaderin C) Pigment epithelium-derived factor / SERPINEF1 and D) Clusterin and many others. Many of these non-collagenous proteins did not show regulation by disease state in this study.
