# Supplementary material for: Tissue and extracellular matrix remodeling of the subchondral bone during osteoarthritis of knee joints as revealed by spatial mass spectrometry imaging
Source: Bone Res. 2026 Jan 26;14:14. doi: 10.1038/s41413-025-00495-0 (PMC12835079; doi:10.1038/s41413-025-00495-0)
Supplement: Supplementary file 11 — Supplementary Figure 11 [file 41413_2025_495_MOESM11_ESM.pptx]

## Slide 1
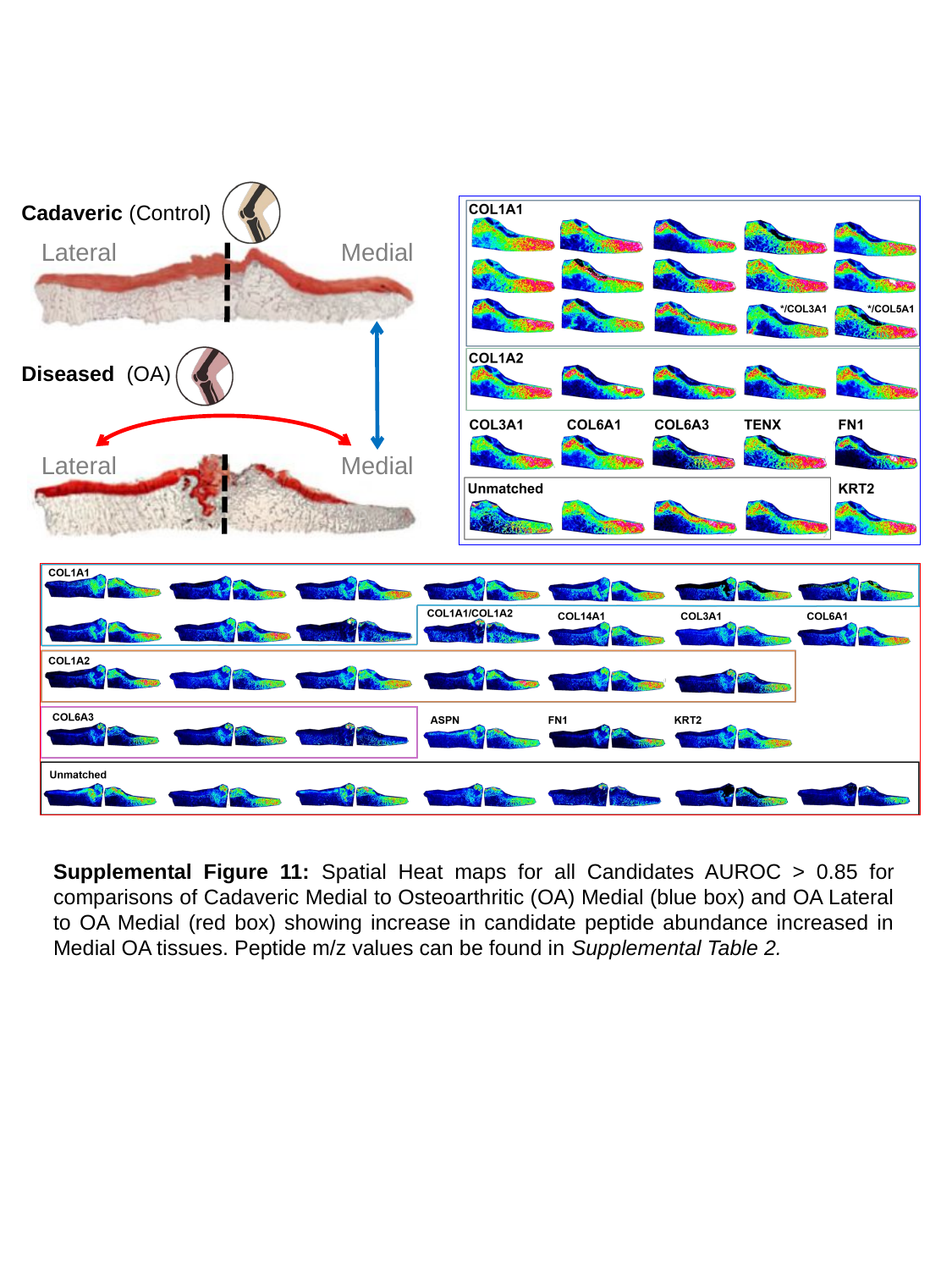

Cadaveric (Control)
Lateral
Medial
Diseased (OA)
Lateral
Medial
Supplemental Figure 11: Spatial Heat maps for all Candidates AUROC > 0.85 for comparisons of Cadaveric Medial to Osteoarthritic (OA) Medial (blue box) and OA Lateral to OA Medial (red box) showing increase in candidate peptide abundance increased in Medial OA tissues. Peptide m/z values can be found in Supplemental Table 2.
