# Supplementary material for: Tissue and extracellular matrix remodeling of the subchondral bone during osteoarthritis of knee joints as revealed by spatial mass spectrometry imaging
Source: Bone Res. 2026 Jan 26;14:14. doi: 10.1038/s41413-025-00495-0 (PMC12835079; doi:10.1038/s41413-025-00495-0)
Supplement: Supplementary file 12 — Supplementary Figure 12 [file 41413_2025_495_MOESM12_ESM.pptx]

## Slide 1
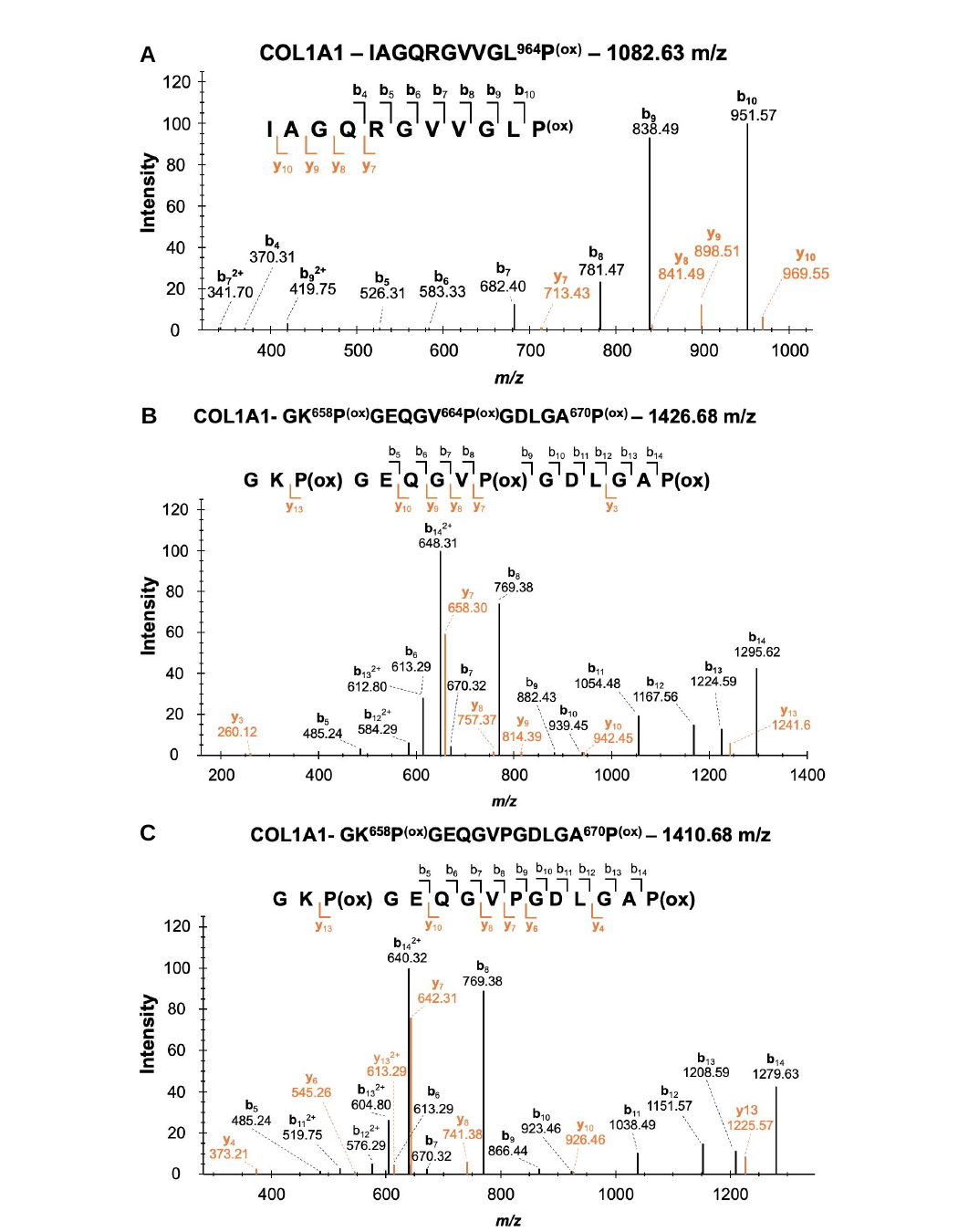

A
B
C

## Slide 2
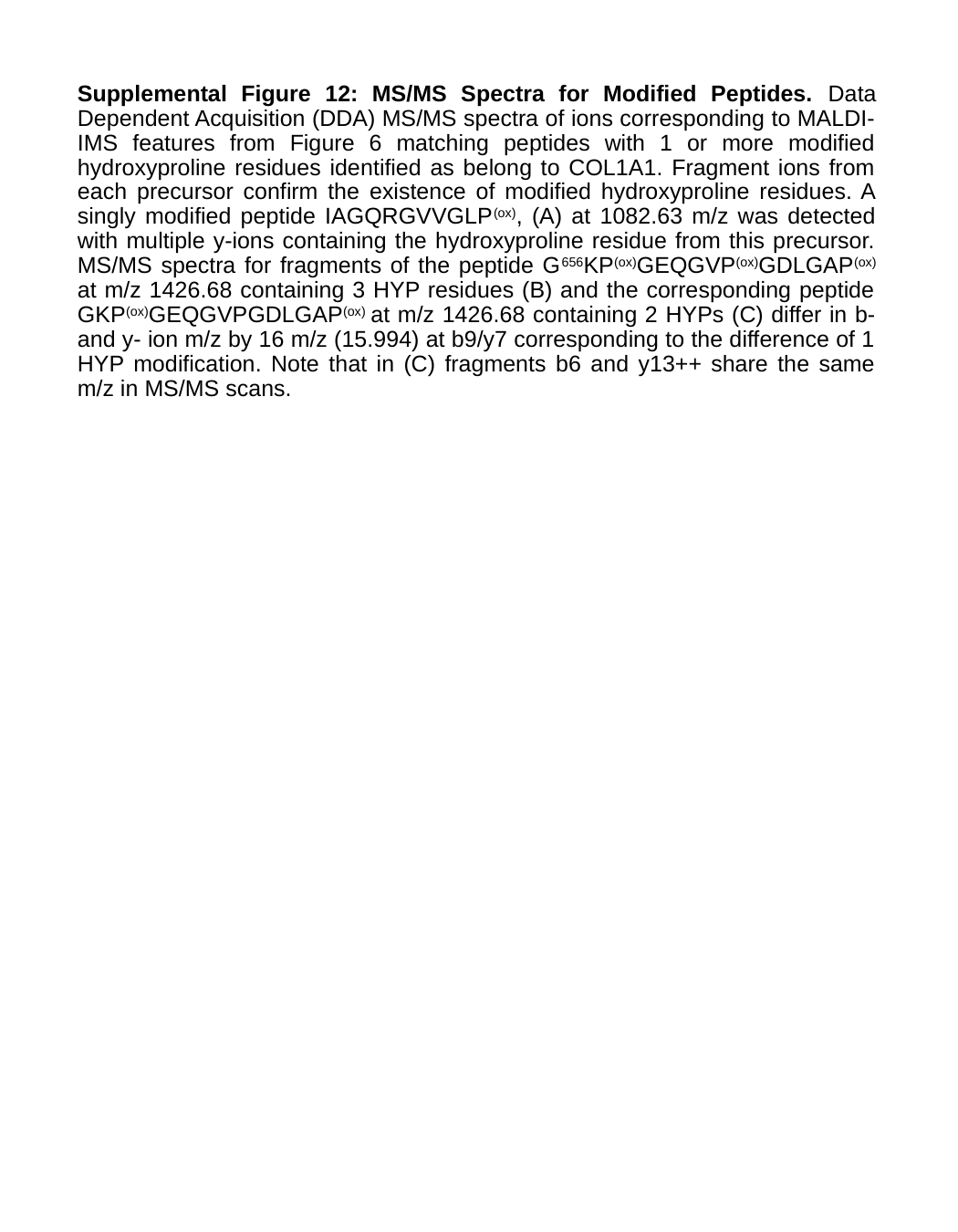

# Supplemental Figure 12: MS/MS Spectra for Modified Peptides. Data Dependent Acquisition (DDA) MS/MS spectra of ions corresponding to MALDI-IMS features from Figure 6 matching peptides with 1 or more modified hydroxyproline residues identified as belong to COL1A1. Fragment ions from each precursor confirm the existence of modified hydroxyproline residues. A singly modified peptide IAGQRGVVGLP(ox), (A) at 1082.63 m/z was detected with multiple y-ions containing the hydroxyproline residue from this precursor. MS/MS spectra for fragments of the peptide G656KP(ox)GEQGVP(ox)GDLGAP(ox) at m/z 1426.68 containing 3 HYP residues (B) and the corresponding peptide GKP(ox)GEQGVPGDLGAP(ox) at m/z 1426.68 containing 2 HYPs (C) differ in b-and y- ion m/z by 16 m/z (15.994) at b9/y7 corresponding to the difference of 1 HYP modification. Note that in (C) fragments b6 and y13++ share the same m/z in MS/MS scans.
