# Supplementary material for: Tissue and extracellular matrix remodeling of the subchondral bone during osteoarthritis of knee joints as revealed by spatial mass spectrometry imaging
Source: Bone Res. 2026 Jan 26;14:14. doi: 10.1038/s41413-025-00495-0 (PMC12835079; doi:10.1038/s41413-025-00495-0)
Supplement: Supplementary file 13 — Supplementary Figure 13 [file 41413_2025_495_MOESM13_ESM.pptx]

## Slide 1
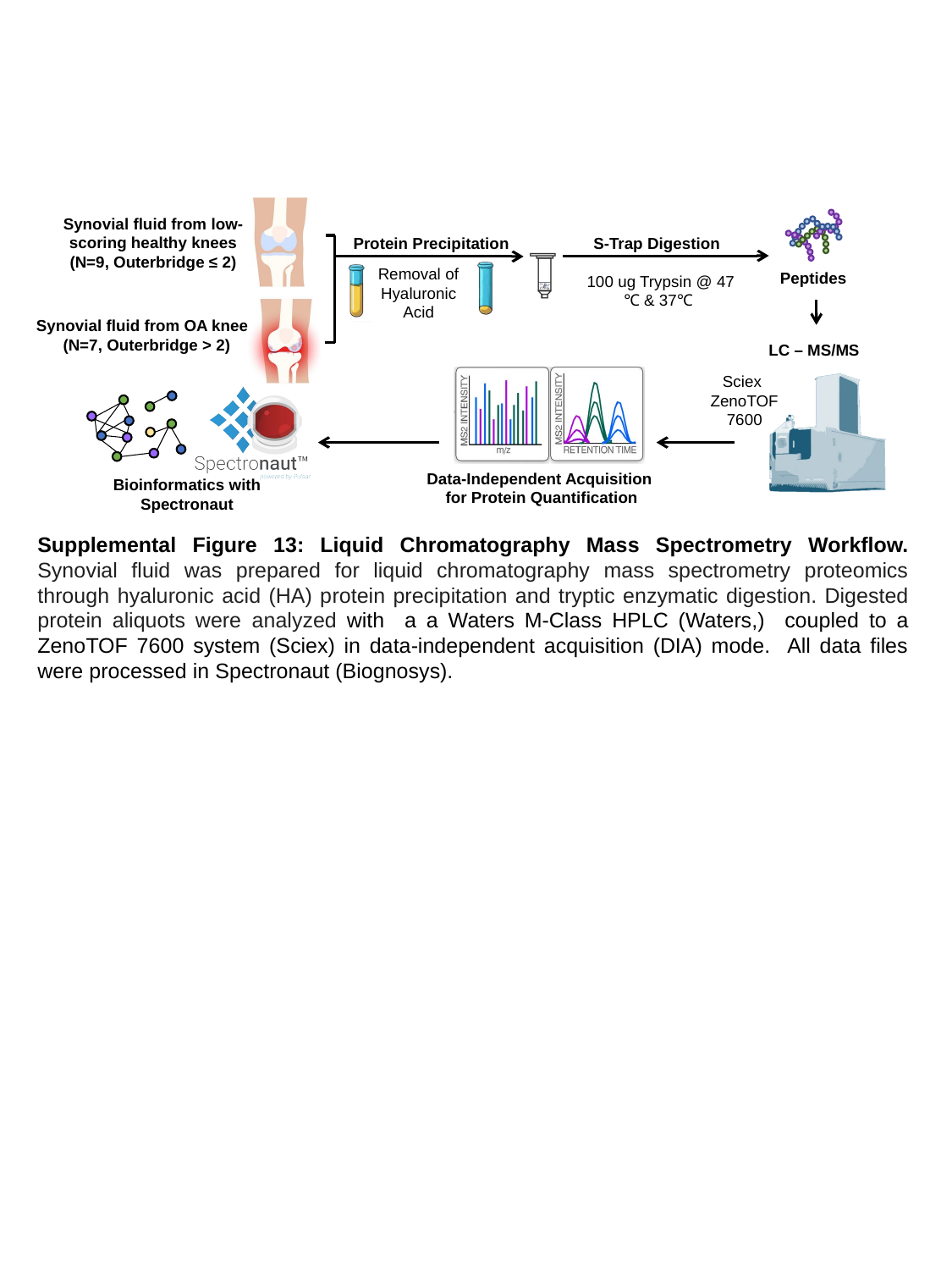

Synovial fluid from low-scoring healthy knees (N=9, Outerbridge ≤ 2)
S-Trap Digestion
Protein Precipitation
Removal of Hyaluronic Acid
Peptides
100 ug Trypsin @ 47 ℃ & 37℃
Synovial fluid from OA knees (N=7, Outerbridge > 2)
LC – MS/MS
Sciex
ZenoTOF 7600
Data-Independent Acquisition
 for Protein Quantification
Bioinformatics with Spectronaut
Supplemental Figure 13: Liquid Chromatography Mass Spectrometry Workflow. Synovial fluid was prepared for liquid chromatography mass spectrometry proteomics through hyaluronic acid (HA) protein precipitation and tryptic enzymatic digestion. Digested protein aliquots were analyzed with a a Waters M-Class HPLC (Waters,) coupled to a ZenoTOF 7600 system (Sciex) in data-independent acquisition (DIA) mode. All data files were processed in Spectronaut (Biognosys).
