# Supplementary material for: Tissue and extracellular matrix remodeling of the subchondral bone during osteoarthritis of knee joints as revealed by spatial mass spectrometry imaging
Source: Bone Res. 2026 Jan 26;14:14. doi: 10.1038/s41413-025-00495-0 (PMC12835079; doi:10.1038/s41413-025-00495-0)
Supplement: Supplementary file 14 — Supplementary Figure 14 [file 41413_2025_495_MOESM14_ESM.pptx]

## Slide 1
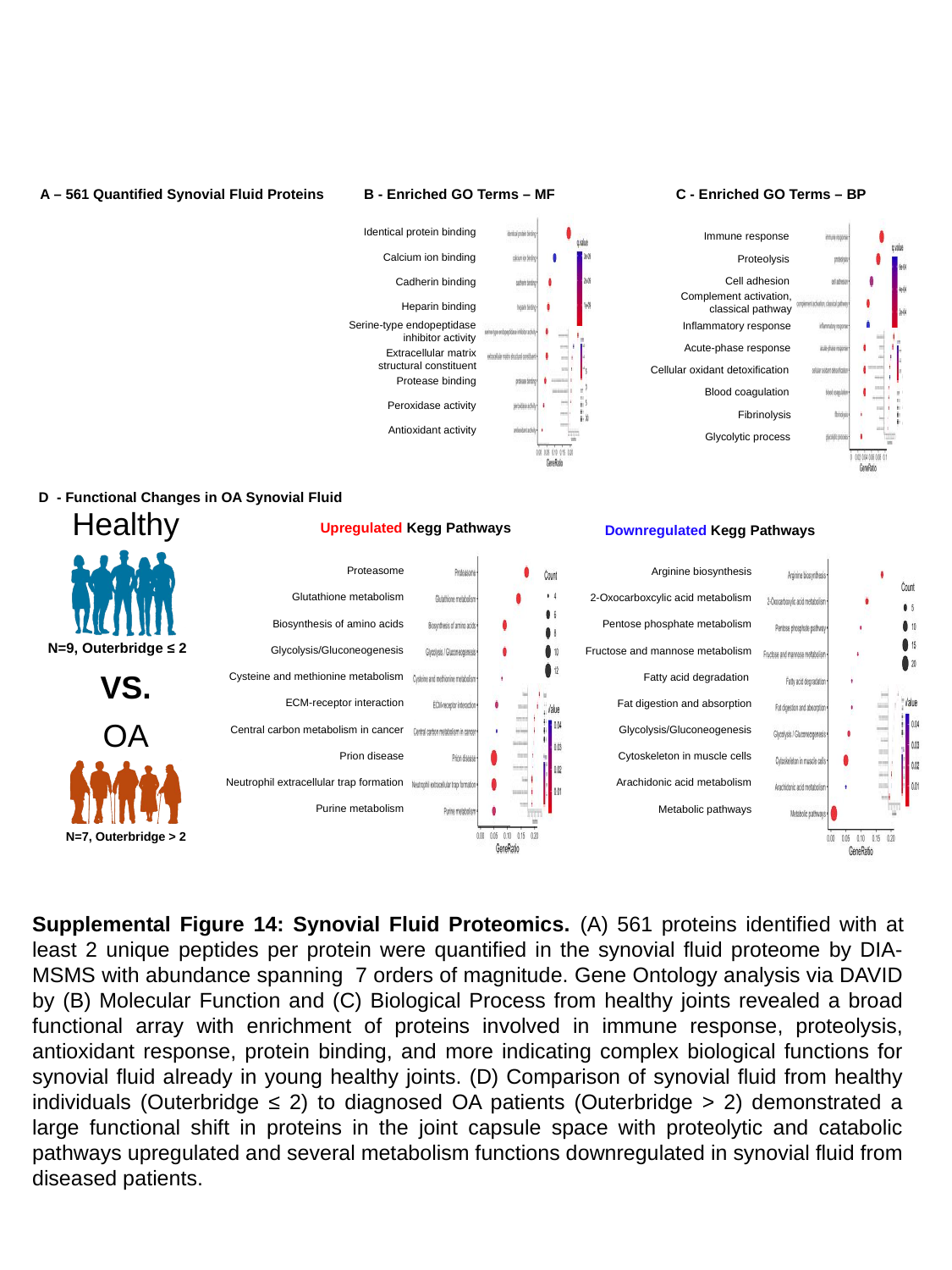

A – 561 Quantified Synovial Fluid Proteins
B - Enriched GO Terms – MF
C - Enriched GO Terms – BP
Identical protein binding
Calcium ion binding
Cadherin binding
Heparin binding
Extracellular matrix
structural constituent
Protease binding
Peroxidase activity
Antioxidant activity
Immune response
Proteolysis
Cell adhesion
Complement activation,
classical pathway
Inflammatory response
Acute-phase response
Cellular oxidant detoxification
Blood coagulation
Fibrinolysis
Glycolytic process
Serine-type endopeptidase
 inhibitor activity
D - Functional Changes in OA Synovial Fluid
Healthy
 Upregulated Kegg Pathways
 Downregulated Kegg Pathways
Proteasome
Glutathione metabolism
Biosynthesis of amino acids
Glycolysis/Gluconeogenesis
Cysteine and methionine metabolism
ECM-receptor interaction
Central carbon metabolism in cancer
Prion disease
Neutrophil extracellular trap formation
Purine metabolism
Arginine biosynthesis
2-Oxocarboxcylic acid metabolism
Pentose phosphate metabolism
Fructose and mannose metabolism
Fatty acid degradation
Fat digestion and absorption
Glycolysis/Gluconeogenesis
Cytoskeleton in muscle cells
Arachidonic acid metabolism
Metabolic pathways
N=9, Outerbridge ≤ 2
VS.
OA
N=7, Outerbridge > 2
Supplemental Figure 14: Synovial Fluid Proteomics. (A) 561 proteins identified with at least 2 unique peptides per protein were quantified in the synovial fluid proteome by DIA-MSMS with abundance spanning 7 orders of magnitude. Gene Ontology analysis via DAVID by (B) Molecular Function and (C) Biological Process from healthy joints revealed a broad functional array with enrichment of proteins involved in immune response, proteolysis, antioxidant response, protein binding, and more indicating complex biological functions for synovial fluid already in young healthy joints. (D) Comparison of synovial fluid from healthy individuals (Outerbridge ≤ 2) to diagnosed OA patients (Outerbridge > 2) demonstrated a large functional shift in proteins in the joint capsule space with proteolytic and catabolic pathways upregulated and several metabolism functions downregulated in synovial fluid from diseased patients.
