# Supplementary material for: Tissue and extracellular matrix remodeling of the subchondral bone during osteoarthritis of knee joints as revealed by spatial mass spectrometry imaging
Source: Bone Res. 2026 Jan 26;14:14. doi: 10.1038/s41413-025-00495-0 (PMC12835079; doi:10.1038/s41413-025-00495-0)
Supplement: Supplementary file 15 — Supplementary Figure 14 [file 41413_2025_495_MOESM15_ESM.pptx]

## Slide 1
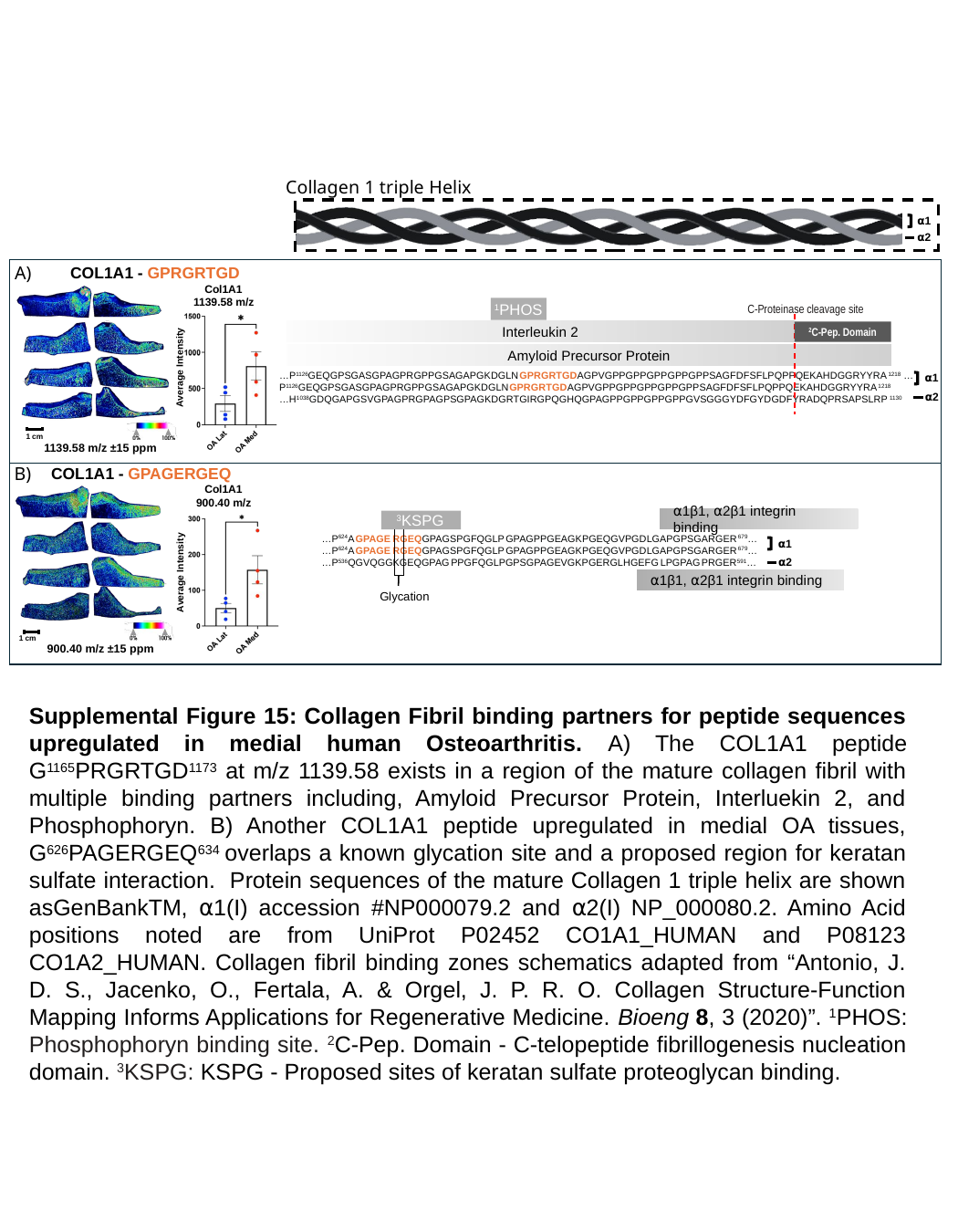

Collagen 1 triple Helix
⍺1
⍺2
A)
COL1A1 - GPRGRTGD
Col1A1 1139.58 m/z
1PHOS
C-Proteinase cleavage site
Average Intensity
Interleukin 2
2C-Pep. Domain
Amyloid Precursor Protein
…P1126GEQGPSGASGPAGPRGPPGSAGAPGKDGLNGPRGRTGDAGPVGPPGPPGPPGPPGPPSAGFDFSFLPQPPQEKAHDGGRYYRA1218 …P1126GEQGPSGASGPAGPRGPPGSAGAPGKDGLNGPRGRTGDAGPVGPPGPPGPPGPPGPPSAGFDFSFLPQPPQEKAHDGGRYYRA1218
…H1038GDQGAPGSVGPAGPRGPAGPSGPAGKDGRTGIRGPQGHQGPAGPPGPPGPPGPPGVSGGGYDFGYDGDFYRADQPRSAPSLRP1130
⍺1
⍺2
1 cm
1139.58 m/z ±15 ppm
B)
COL1A1 - GPAGERGEQ
Col1A1 900.40 m/z
3KSPG
Average Intensity
⍺1β1, ⍺2β1 integrin binding
…P624A GPAGE RGEQGPAGSPGFQGLP GPAGPPGEAGKPGEQGVPGDLGAPGPSGARGER679…
…P624A GPAGE RGEQGPAGSPGFQGLP GPAGPPGEAGKPGEQGVPGDLGAPGPSGARGER679…
…P536QGVQGGKGEQGPAG PPGFQGLPGPSGPAGEVGKPGERGLHGEFG LPGPAG PRGER591…
⍺1
⍺2
⍺1β1, ⍺2β1 integrin binding
Glycation
1 cm
900.40 m/z ±15 ppm
Supplemental Figure 15: Collagen Fibril binding partners for peptide sequences upregulated in medial human Osteoarthritis. A) The COL1A1 peptide G1165PRGRTGD1173 at m/z 1139.58 exists in a region of the mature collagen fibril with multiple binding partners including, Amyloid Precursor Protein, Interluekin 2, and Phosphophoryn. B) Another COL1A1 peptide upregulated in medial OA tissues, G626PAGERGEQ634 overlaps a known glycation site and a proposed region for keratan sulfate interaction. Protein sequences of the mature Collagen 1 triple helix are shown asGenBankTM, ⍺1(I) accession #NP000079.2 and ⍺2(I) NP_000080.2. Amino Acid positions noted are from UniProt P02452 CO1A1_HUMAN and P08123 CO1A2_HUMAN. Collagen fibril binding zones schematics adapted from “Antonio, J. D. S., Jacenko, O., Fertala, A. & Orgel, J. P. R. O. Collagen Structure-Function Mapping Informs Applications for Regenerative Medicine. Bioeng 8, 3 (2020)”. 1PHOS: Phosphophoryn binding site. 2C-Pep. Domain - C-telopeptide fibrillogenesis nucleation domain. 3KSPG: KSPG - Proposed sites of keratan sulfate proteoglycan binding.
